# A Symmetry‐Based Kinematic Theory for Nanocrystal Morphology Design

**DOI:** 10.1002/anie.202200753

**Published:** 2022-03-14

**Authors:** Bing Ni, Guillermo González‐Rubio, Felizitas Kirner, Siyuan Zhang, Helmut Cölfen

**Affiliations:** ^1^ Physical Chemistry University of Konstanz Universitätsstrasse 10 78457 Konstanz Germany; ^2^ Max-Planck-Institut für Eisenforschung GmbH Max-Planck-Strasse 1 40237 Düsseldorf Germany

**Keywords:** Au Dendrimers, Kinematic Theory, Morphology Evolution and Design, Symmetry Breaking

## Abstract

The growth of crystalline nanoparticles (NPs) generally involves three processes: nucleation, growth, and shape evolution. Among them, the shape evolution is less understood, despite the importance of morphology for NP properties. Here, we propose a symmetry‐based kinematic theory (SBKT) based on classical growth theories to illustrate the process. Based on the crystal lattice, nucleus (or seed) symmetry, and the preferential growth directions under the experimental conditions, the SBKT can illustrate the growth trajectories. The theory accommodates the conventional criteria of the major existing theories for crystal growth and provides tools to better understand the symmetry‐breaking process during the growth of anisotropic structures. Furthermore, complex dendritic growth is theoretically and experimentally demonstrated. Thus, it provides a framework to explain the shape evolution, and extends the morphogenesis prediction to cases, which cannot be treated by other theories.

## Graphical abstract

A symmetry‐based kinematic theory (SBKT) based on classical growth theories to illustrate shape evolution is proposed. Based on the crystal lattice, nucleus symmetry, and the preferential growth directions under the experimental conditions, the SBKT can illustrate the growth trajectories of NPs. The theory accommodates the conventional theories for crystal growth and provides tools to better understand the symmetry‐breaking.

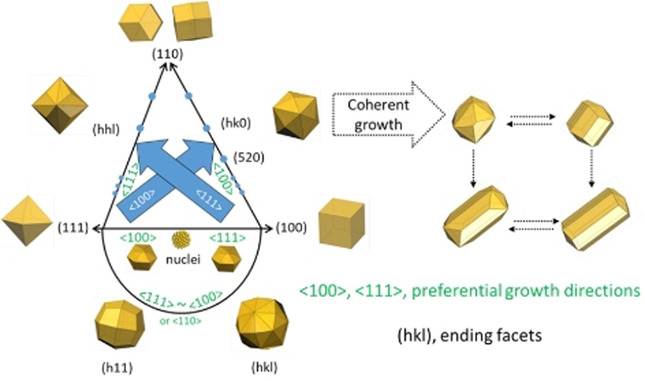

## Introduction

Understanding nanocrystal shape evolution is fundamental to many key questions in nanoscience. These include retrosynthetic analysis and designed synthesis of nanoparticles,^[1]^ dendritic growth inhibition of metal batteries during charging,^[2]^ how NPs reshape or degrade during catalysis,^[3]^ and so forth. However, most conventional methods are mainly based on the morphology‐time profile, acquired by in situ,^[4]^ in operando,^[5]^ ex situ,^[6]^ or theoretical simulations.^[7]^ More specifically, the energy and stability of the exposed facets are usually the primary concern, that stable facets remain and unstable facets disappear.[^8^, ^9^] Unfortunately, this criterion does not provide details about the atom‐by‐atom growth mechanism and cannot answer why cubes can evolve into cuboids, for example.[^10^, ^11^]

The growth of nanoparticles generally involves three processes: nucleation, growth, and shape evolution. Different theories have been developed to treat the corresponding phenomena. On the one hand, in classical figures, crystal growth is enabled by adding building units in a one‐by‐one manner, and the morphology is the result of the interplay between the nucleation process and the movement of atomic terraces or facets (kinematic theory). In general, after the initial nucleation of a new crystal phase, growth can proceed via the emergence of an atom layer (2D nucleation) on its surface that can later advance. It is worth mentioning that the layer advancement proceeds via both the deposition of new building blocks from solution and the surface atom diffusion to the kink sites (or half‐crystal sites, in other words). In an alternative pathway, growth can be controlled by the so‐called screw‐dislocation‐driven growth, which provides a non‐vanishing step site and bypasses the 2D nucleation process. These processes were summarized by Burton, Cabrera, and Frank, and named as BCF theory.^[12]^

On the other hand, the shape evolution can be described by Frank's kinematic theory,[^13^, ^14^] mainly considering slow‐growing facets. The trajectory of the shape evolution can be predicted using a polar diagram of slowness vectors (which is impossible to obtain experimentally). Although the theory is mathematically rigorous, the polar diagram is hardly suitable for predicting the shape evolution of real systems due to its complexity and the unavailability of the growth rates in experimental conditions. Nonetheless, the concept that slow‐growing facets (also corresponding to stable facets or low energy facets) are preserved is used as a conventional idea to explain nanocrystal morphology evolution. Within this framework, the advancement of nanoscience and nanochemistry has paved the way for an array of crystalline NPs with distinct morphological features, thereby allowing for greater knowledge of growth mechanisms.^[15]^ Nevertheless, understanding the shape evolution from an atom‐by‐atom manner is still challenging, and, as a result, it has not been advanced so far.

Here, we propose the SBKT to illustrate the shape evolution process of crystalline NPs. We keep the classical assumption that atomic building blocks deposit onto the nuclei (or seeds) in a one‐by‐one manner. Surface 2D nucleation and layer advancement are considered as the methods to enlarge the seeds, and the shape evolution is defined by the collective behavior of the two processes. By considering symmetry and preferential growth directions (PGDs), the kinematic theory can be radically simplified and eventually becomes applicable to real cases. By considering the properties of kinematic waves, which are not included in the classic theories, the growth trajectory can be better comprehended. We confirmed the convenience of the SBKT to understand the seed‐mediated growth of Au NP with complex anisotropic structures resulting from the occurrence of multiple symmetry‐breaking events during growth. We chose a seed‐mediated growth route because it is a good platform to study the shape evolution of NPs since the structure of the nuclei (seeds) is well‐defined.[^16^, ^17^] Combining the PGD with the symmetry of the seed and lattice, we are able to explain the symmetry breaking to form nanorods during growth. The process is called “coherent growth” which means the kinematic wave induced by a 2D nucleation process could alter the structures of the adjacent nucleation sites, making such positions different (i.e., altering nucleation rates at such sites). Furthermore, we demonstrate that the SBKT can be used to provide a clear atomic image of the growth of dendritic nanostructures, which are common self‐similar structures in Nature. The reason was ascribed to the presence of several corner sites on seeds which cannot dissipate the kinematic waves rapidly. Accordingly, we afford the synthesis of various Au dendrimers with different generations. These processes cannot be adequately explained by conventional mechanisms such as the stability of facets, Wulff constructions, deposition‐diffusion concepts, etc. Thus, SBKT provides a new perspective to understand the morphology evolution of NPs, which could significantly contribute to the advancement of retrosynthesis methods for NPs.

## Results and Discussion

### SBKT

**Figure 1:**
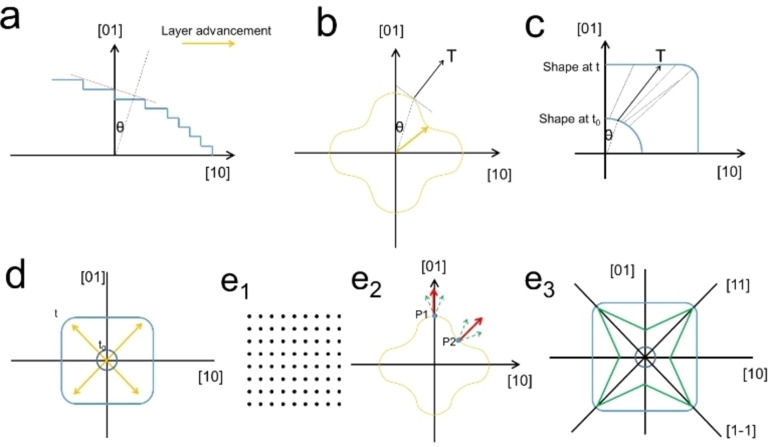
Simplified 2D illustrations sketching the procedures to track a crystal growth trajectory according to Frank's kinematic theory (a–c) and the symmetry‐based kinematic theory (d, e).

Frank's kinematic theory proceeds as follows (Figure 1a–c): a) to evaluate the development of a vicinal facet (red line) in a crystal (blue line), the normal of the facet and the corresponding angle *θ* are marked first, and b) then the polar diagram of the reciprocal of the growth velocity of all orientations (all vicinal facets) has to be estimated or determined (orange line). The point on the polar diagram with an angle of *θ* needs then to be found, and the normal of this point should be marked (T). The orange arrow here indicates the fastest growth direction. The third step c) is to plot the trajectory in real space. The growth trajectory of the site on the surface with an angle of *θ* always points in the direction of T. Doing the same analysis on all surface sites (such as the solid black lines) will lead to an explicit growth trajectory of the shape evolution. This is the geometric expression of the theory without detailing the physical processes. The theory is mathematically rigorous, which is proven by coupling many ordinary and partial differential equations as well as many boundary conditions. However, this method is complicated and hardly applicable in practice because determining the detailed growth rates in real cases is radically challenging (the growth rates might also change during the growth).

**Figure 2:**
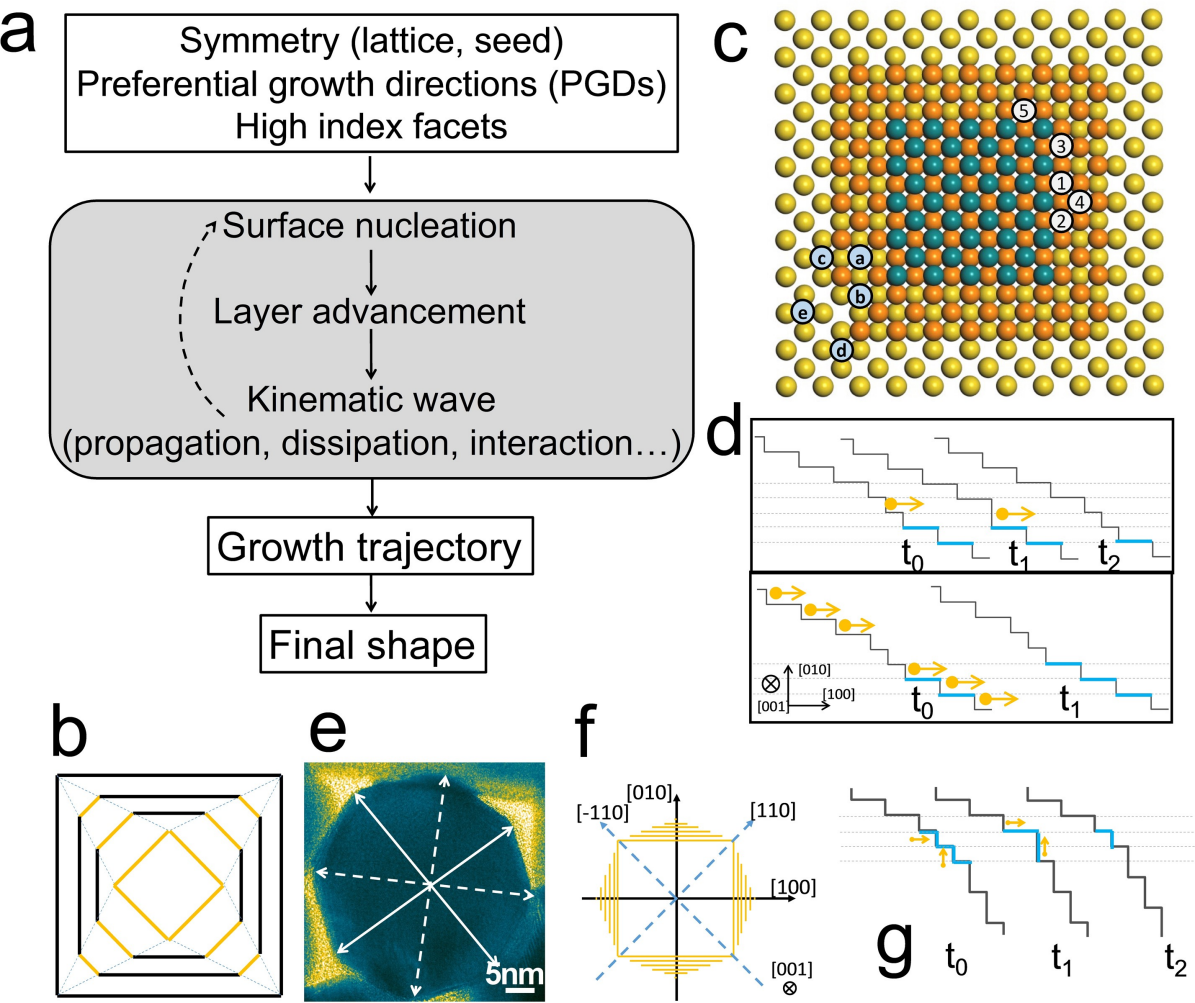
Illustration of the SBKT. a) Logic of using SBKT to predict the NP shape. b) The evolution of inset orange square to the outside black square. c) 1D movement of non‐uniformity. In fcc metals, the first coordination number is 12. The number of nearest bonds when an atom deposits at site a, b, c, d, e is 8, 7, 6, 5, 4, respectively, while the number of dangling bonds is 4, 5, 6, 7, 8, respectively. d) 2D movement of non‐uniformity. The non‐uniformity can propagate to the right side (up) if the one‐atom step advances faster than the multi‐atom step or move to the left side (down) if the one‐atom step advances slowly. e) HRTEM image of a standing Au NR. The white solid arrows show the <100> directions, while the dashed arrows show the <110> directions. The directions were drawn with the help of fast Fourier transform patterns. f) Cross section of an Au NRs. g) Non‐uniformity would disappear at the <110> edges in the growth of Au NRs.

To simplify the analysis of NP shape evolution during the growth process, the proposed symmetry‐based kinematic theory introduces the concept of PGDs (Figure 1d, orange arrows). For instance, in the case of a 2D crystal with a highly symmetric circular shape (Figure 1d, dark blue) and a simple square lattice (e1), if the PGDs are <11> (orange arrows), the original NP shape evolves to a square (Figure 1d, blue). Detailed illustration of this process considers that (e1) the 2D lattice has the symmetry elements *C*_4_, *m,* $\bar{1}$ ; and (e2) the resulting polar diagram also possesses such symmetry elements. Thus, those normals of P1 and P2, which are at the intersection of <01> and <11> directions and the polar diagram, should be along with the corresponding <01> and <11> directions (red arrows). Otherwise, there would be two normals of one point (green arrows) according to symmetry considerations, which is impossible. The growth trajectories of points at <01> and <11> directions (e3) would always follow these directions (black lines). If high index facets are not efficiently stabilized by the growth environment, the shape would eventually evolve from the circle to the square. However, when high index facets can be stabilized during growth, a star‐like morphology would be finally adopted (green). Indeed, the stability of high index facets can be inferred from the relative growth velocities between <01> and <11> directions. Hence, while fast growth directions need to be known, there is no need to determine the growth velocity values. As a result, the growth trajectory can be simply described by the PGDs (i.e., fast growth directions) with the help of the stability of high index facets (Figure 2a). So far, we have proven that only the PGDs and the lattice symmetry need to be considered to hold the entire content of the rigorous classic kinematic theory. It is worth noting that described analysis could be extended to any crystalline structure.

The outcome of focusing on the PGDs or the stable facets is practically the same. The facet will disappear if the growth in a given direction is too fast. In other words, this is where the growth is most likely occurring, and the exposed facets result from the layer advancement process of the disappearing facets. For example, in a 2D illustration (Figure 2b), the small orange square's evolution to the big black square can be interpreted as either fast growth of orange edges or slow growth of black edges. The atom deposition would occur on the orange edges at a higher rate, thereby leading to faster nucleation and layer advancement processes. Thus, their growth occurs along the PGDs, and the layer advancement would stop at the black edges due to their higher stability (i.e., lower free surface energy), and they remain after growth. In summary, the growth is mainly occurring on the orange square, and their evolution would eventually end up with the black square. Thus, the surface with the highest energy would grow fast, and the directions perpendicular to such a surface are the PGDs. Considering the symmetry analysis shown in Figure 1e, only the surface energies of facets, which lie on the symmetry element of mirror planes, need to be considered.

The fundamental physical processes of the classical kinematic theory rely on the concept of kinematic waves. In real growth processes, one layer might catch up with or fall behind another layer due to a new nucleation event, the presence of impurities, or other reasons, producing some non‐uniformities in the advancement of steps. The position of the non‐uniformity can change during growth, and their displacement can be treated as a kinematic wave (Figure S1a). The kinematic wave is the collective behavior of the advancement of different layers, and it can effectively propagate non‐uniformities to other regions. The mathematical treatment of the propagations led to the geometric expression of the theory. It is worth noting that the classical kinematic theory was developed before the burgeoning of nanochemistry. Thus, the kinematic waves at different positions were deemed to propagate independently on large enough planes to eventually dissipate all the non‐uniformities. The properties of kinematic waves at the nanoscale have not been discussed yet, which might hold the key to understanding the shape evolution of NPs.

Before going into details, we would like to sort out the logic of interpreting the growth trajectory. The hierarchy of the concepts in the SBKT is (level 1) shape evolution; (level 2) PGDs and kinematic wave; (level 3) surface nucleation and layer advancement; (level 4) atom deposition and surface atom diffusion. The processes at concept level 3 and level 4 have been treated by BCF theory, and we further clarify and improve the concept levels 2 and 1 in the classical kinematic theory. Atom deposition and surface diffusion are the fundamental growth processes. The formation of a surface nucleus indicates that the surface adatoms at this area are stable. The layer advancement would be initiated by a surface nucleation event. The two processes could proceed via both atom deposition and surface nucleation. The PGDs are determined by the surface nucleation and layer advancement rates at different facets, and the kinematic wave is the collective behavior of layer advancements. The shape evolution is at the top level, and to illustrate it, it is just needed to focus on the PGDs and the properties of the kinematic wave (i.e., without a detailed study of the atom deposition and surface atom diffusion behavior). Altogether, the growth trajectory would be defined by the PGDs, and modified by the properties of kinematic waves under the corresponding growth conditions.

Now we aim at discussing the properties of kinematic waves at the nanoscale (Figure S1b). In this scenario, to provide a better understanding of non‐uniformity propagation with an atomic view, we introduce the concept of half‐crystal sites,[^18^, ^19^] which considers that the number of forming bonds is equal to the dangling bonds (such as Figure 2c, site‐C, in an fcc metal). Atoms at these sites have an equal possibility for atom attachment and detachment. Thus, atoms at these sites would be in a dynamic equilibrium of attachment and detachment. Atoms located at sites with a larger number of dangling bonds would tend to detach (special note: the atom detachment can also be viewed as the surface atom diffusion since the probability of this atom moving to other surface areas is more significant than that of dissolving). This simple probability comparison can help us to understand the driving force for layer advancement without getting bogged down in overly precise analysis in a specific given case. For example, in a 1D propagation process (such as that of the blue atom layer's advancement depicted in Figure 2c), an atom located at site‐1 would lock the two adjacent blue atoms and thereby facilitate the growth along the directions from site‐1 to 2 and site‐1 to 3 due to the enhanced probability of atom attachment. For the same reason, the layer would advance to site‐4, although at a slower rate than from site‐2 to 3 (i.e., due to their more favorable coordination environment). When an atom deposits at site‐3, the locked corner blue atom would facilitate the deposition at site‐5. The described analysis implies that the layer advancement would automatically propagate to other sides of a layer even if its progression only occurred on the right side. Notably, the layer displacement can be understood as the movement of steps when seen from a projection angle (Figure 2d). Hence, in a 2D case, if one‐atom step moves faster than the multi‐atom steps, the non‐uniformity would merge and produce a bilayer‐two‐atom step. However, its movement requires a higher atom flux compared to single‐layer steps, and it would eventually decompose into two single‐layer steps. This analysis indicates that the non‐uniformity could move during growth, a phenomenon that can be seen as the kinematic wave propagation during growth. Notably, this concept is also extrapolated to 3D cases (Figure S1b).

**Figure 3:**
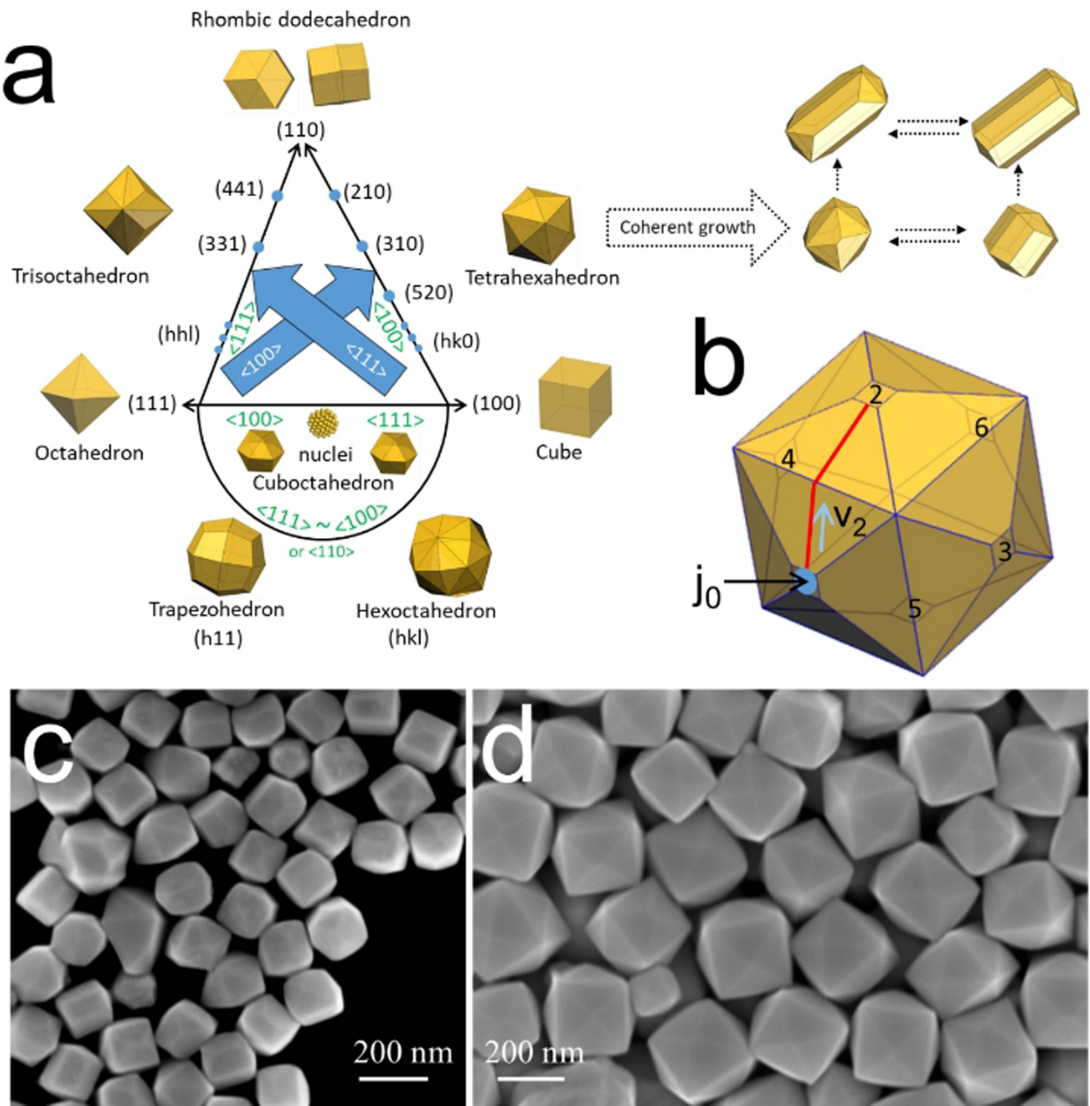
Growth trajectory prediction and symmetry breaking. a) The growth trajectories of different shapes. The orientations (green) in the graph indicate the PGDs, while the facets (black) are the exposed facets. Both the PGDs and the exposed facets are determined by the growth conditions. The shapes before (left side) and after (right side) coherent growth have a symmetry of 4/*m* $\bar{3}$ 2/*m* and 4/*m* 2/*m* 2/*m*, respectively. This diagram illustrates the possible shape evolution from spherical nuclei to different possible optimized morphologies. b) The position of nucleation sites of the THH particle with PGDs of <100>. The nucleation rate, kinematic wave propagation, and distance between different nucleation sites can be denoted as *j*_0_ (s^−1^, blue cycle), *v*_2_ (nm s^−1^, blue arrow), and *L* (nm, red line). c) and d) are the scanning electron microscopy images of the symmetry broken structures and symmetry retaining structures, respectively.

The dissipation of kinematic waves depends on the experimental growth conditions and the stability of facets. Thus, to investigate the suitability of the SBKT for understanding the growth and shape evolution of crystalline NPs, we applied it to investigate the growth of anisotropic Au NPs. In this regard, we benefit from a recently reported method for the synthesis of high quality Au NRs with a controlled anisotropy degree (Figure S2).^[20]^ The main advantage of this method in studying the kinematic wave dissipation is that it relies on the use of small Au NRs as seeds, and therefore the symmetry‐breaking event does not need to be considered. We only need to focus on the enlargement of a preformed rod (symmetry breaking will be considered in detail later). Moreover, the PGDs could be readily determined by geometric analysis. For instance, high‐resolution transmission electron microscopy (HRTEM) images of the standing NRs revealed that the exposed side facets lie perpendicular to the <1+√2 1 0> direction, which bisects the angle between <100> and <110> directions (Figures 2e and S3).[^21^, ^22^] Thereby, the PGD at such synthesis conditions (see Supporting Information) could be assigned to the <100> directions (Figures 2f and S4), and the cross‐section would have the projection of a regular octagon (Figure 2f). Since the layer advancement would end up with high index facets according to the HRTEM results (Figure S3), the non‐uniformity in this case might be caused by the formation of one‐atom steps (Figure 2g). If two of these non‐uniformities from different side facets meet at the edges pointing to the <110> directions (called <110> edges hereafter), the non‐uniformity would disappear and eventually result in the growth of NRs with octagonal cross‐section (Figures 2g and S5a–c). If the non‐uniformities meet at the <100> edges, a new terrace forms, and a new layer can be generated due to the 2D nucleation (Figure S5d–f). This fact suggests that the kinematic wave dissipates at <110> and <100> edges, leading to Au NRs with an octagonal section. The propagation and dissipation of kinematic waves could automatically lead to a structure conforming to the symmetry of the lattice and seeds (Figure S5g–i). Altogether, the final shape of a NP could be predicted by considering the lattice and seed symmetry, PGDs, the stability of high index facets, and the properties of kinematic waves (propagation, dissipation). The PGDs and stability of high index facets are solely dependent on the growth environments and would not change with the shape of seeds. Such information can be obtained by pre‐experiments, or simply from literature. Although the PGDs should be related to the type of surfactants, precursor concentrations, ligands, etc. under the given conditions, there is no need to investigate in detail how these parameters could influence the PGDs. Just the PGDs need to be known according to pre‐experiments or from the literature, and by using the PGDs, the shape evolution can be inferred. Furthermore, the PGDs stay stable even if the concentration of reactants and temperature change a lot in the Au NR investigated system (Figure S6). If the kinematic waves at different facets propagate and dissipate independently, the growth trajectory in a fcc lattice can be predicted as shown in Figure 3a according to the PGDs.

### Symmetry Breaking

On the nanoscale, different kinematic waves might interact during the growth of NPs possibly leading to symmetry breaking. Here we investigated the shape evolution of various seeds at the same growth conditions with the evolution of Au NRs to illustrate how and why the symmetry is broken according to the SBKT. The Au crystal lattice (fcc) has a symmetry of 4/*m* $\bar{3}$ 2/*m*. If the growth can maintain the symmetry of the lattice, corresponding morphologies can be predicted (Figure 3a). The shapes have all been experimentally obtained with fcc lattices^[23]^ (although not all within Au). Here, it is worth mentioning that Wulff constructions typically employed to understand NP morphology evolution during growth could only cover a part of these shapes (mainly the cuboctahedron or truncated structures, also included in Figure 3a). The Wulff construction predicts the equilibrium shape of a particle by considering the balance between the total surface area and the energy of the exposed facets. This theory is an extension of that of Gibbs and Curie, and it is also known as Gibbs–Curie–Wulff's theorem. More specifically, it states that in equilibrium, the distances of the crystal faces from a point within the crystal (called Wulff's point) are proportional to the corresponding specific surface energies of these faces. This implies that such energies should be determined and used as input to predict the preferred morphology of the NP, which is practically impossible in most NP systems (computational modeling is typically required to gain insight into surface free energies for NP exposed facets). Moreover, as it mainly considers the thermodynamic aspects of the NP growth, it fails to predict morphologies determined by kinetic parameters such as the relative rates of atom deposition and surface diffusion or how and where atoms deposit. In fact, structures with morphologies not predicted by Wulff′s theorem can be obtained depending on the experimental growth environments.

Starting from cubic NPs as an example here, they can evolve to tetrahexahedra (THH) by forming pyramids at the 6 facets due to the PGDs of <100> directions in this synthesis. To maintain the symmetry, all the nucleation sites should evolve in an independent manner. For THH obtained under this condition, there would be 6 equivalent nucleation sites located at the tip of the pyramids due to the PGDs of <100> directions (Figure 3b). The nucleation rate, kinematic wave propagation, and distance between different nucleation sites can be denoted as *j*_0_ (s^−1^), *v*_2_ (nm s^−1^), and *L* (nm), respectively. Once a nucleation event occurs at site‐1, the kinematic wave would start to propagate. If the kinematic wave can reach the adjacent nucleation sites, such as site‐2 (as well as site‐3,4,5), before the next nucleation event occurs (the advent of a second kinematic wave), then this kinematic wave would change the structure of site‐2 (as well as site‐3,4,5), thereby diverging from that of site‐1 and site‐6 (Figure S7). Meanwhile, if a new surface nucleation event occurs before the complete dissipation of this kinematic wave, the probabilities of nucleation at site‐1/6 and site‐2/3/4/5 are different. Thus, the NP symmetry would be broken (Figure S8a, c, d). The resulting NP symmetry would be 4/*m* 2/*m* 2/*m*. Since the primary function of the kinematic wave is to dissipate non‐uniformities in the nanocrystal, if the first kinematic wave has propagated through the NP (the time required for the complete dissipation is denoted as *t*_rela*x*_), the structures of site‐1, site‐2, etc., would become equivalent again. Hence, the symmetry is broken when *t*_rela*x*_>1/*j*_0_>*L*/*v*_2_ due to the coherence of the adjacent nucleation sites and several kinematic waves (the second nucleation process would initiate a second kinematic wave). This growth mode, named coherent growth, would lead to a symmetry‐breaking process while all the exposed facets are still equivalent. The symmetry would be further reduced with more severe coherent growth (Figure S8b).

To demonstrate further the role of coherent growth on the formation of anisotropic structures, we investigated the shape evolution of cubes with different dimensions under Au NR synthesis conditions (i.e., all growth conditions were the same except for the use of Au cubes as seeds). It is expected that by increasing the cube dimension, the symmetry‐breaking process will be less favored, as the adjacent nucleation sites would be distant (L is larger) and would grow independently, which helps to maintain the THH symmetry (Figures 3b and S9). Indeed, we could observe this phenomenon when ≈110 nm cubes were used as seeds, while symmetry reduction was noticed when ≈24 nm Au cubes were utilized for seeding due to coherent growth (L is small) (Figures 3c, d and S9a, b). Some elongated particles in Figure 3c are close to the shape of conventional Au NRs (Figure S10).^[24]^ Notably, similar results were obtained using Au nanooctahedra or Au nanocuboctahedra as seeds (Figure S9c, d). These results suggest that the seed morphology has relative importance when the magnitude of the growth is large (i.e., greater differences between the seed and the product sizes), since the final shape would be defined by the PGDs and modified by the extent of coherent growth. The seed‐size‐dependence of the symmetry breaking process in the Au NRs formation has been widely investigated, as only seeds with dimensions below 4 nm can evolve into Au NRs.^[25]^ The SBKT provides a general and simple figure to understand all these shape evolutions, while other theories generally need to adopt many specific and non‐transferrable assumptions (Figure S11).^[26]^

Overall, SBKT methodology can help provide insight into the origin of symmetry‐breaking events in crystalline NPs, which should eventually allow us to understand the NP morphology design principles better and retrospect the reported phenomena (Figure S12). For example, when the growth rates along <100> and <111> directions are comparable, sphere‐like NPs could be preferentially formed^[27]^ (which cannot be adequately explained by Wulff constructions or the stabilization of certain facets, since spheres do not show preferred faceting). Moreover, sphere‐like NPs with fcc lattice could evolve to cubes or octahedra depending on the relative growth rates along corresponding directions, which could be experimentally obtained by tuning the growth conditions (e.g., temperature, precursor concentrations or surface ligand concentration, among others). Notably, such phenomena cannot be easily dealt with using other theoretical methods, while the SBKT provides a simple criterion to explain them. If coherent growth occurs, the formation of nanostructures with reduced symmetry could be expected. Altogether, experimental researchers could predict the morphogenesis by designing the experimental PGDs and controlling the degree of coherent growth.

### Controlled Synthesis of Au Dendrimers

**Figure 4:**
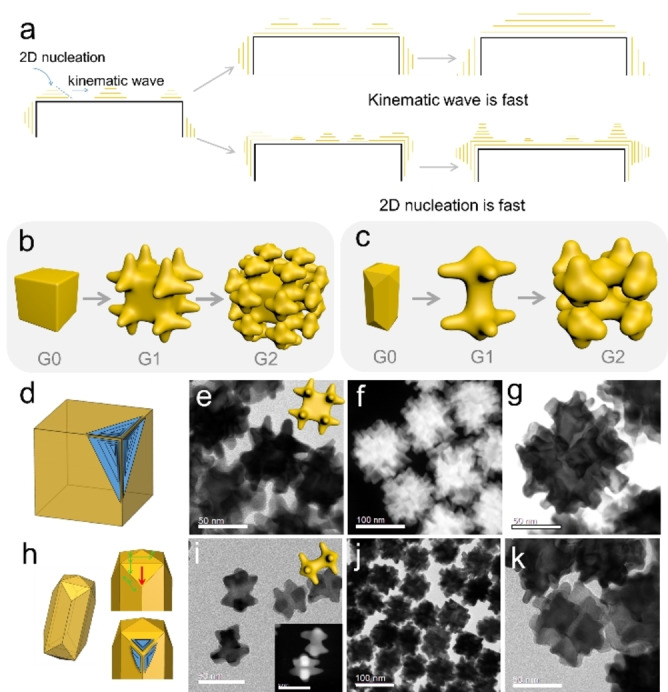
Dendritic growth of Au NPs with PGDs of <100> directions. a) Scheme to illustrate the situations that the kinematic wave or 2D nucleation dominate the process. b, c) Dendrimer formation by using cubes or rods as seeds. d) Sites on an Au nanocube where atoms could accumulate. The blue triangle platform indicates the position of fast 2D nucleation and atom accumulations, which can serve as the site for dendritic growth. e) TEM image of the Au nanocube G1 dendrimers. f, g) Scanning TEM (STEM) image of the Au nanocube G2 dendrimers. h) The shape of the 24‐facet NRs, and the movement of the kinematic waves. i) TEM image of the Au NR G1 dendrimers. Inset shows the STEM image. j, k) TEM images of the Au NR G2 dendrimers. Scale bar: (e, g, i, inset i, k), 50 nm; f, j) 100 nm.

Complex shapes could also be appropriately designed according to the SBKT, such as dendritic‐like NPs (Figure 4). Researches on dendritic growth in material technology concerns mainly temperatures around the melting point of the material when the surface is rough, and extra 2D nucleation is avoided during growth.^[28]^ In this case, atoms can accumulate at any position, and the theories potentially used to explain it only need to consider the mass transport and the total surface energies (e.g., the diffusive transport theory of Ivantsov).^[29]^ Nevertheless, the synthesis of dendritic noble metal NPs is typically carried out at a temperature far below their melting point, where the NP surface should be smooth, and atoms cannot accumulate at any position due to 2D nucleation energy barriers.[^30^, ^31^] According to the analysis of the kinematic wave dissipation in our current growth environments, any non‐uniformities moving to <100> and <110> edges would disappear (Figure S5), while those located at other positions would propagate to the dissipation areas. However, if the nucleation is too fast (e.g., by increasing precursor concentration, due to the exponential relationship between nucleation and supersaturation), atoms would accumulate at positions that cannot dissipate the non‐uniformity fastly. In that case, the formation of dendrites according to the PGDs could be favored.

Taking an Au nanocube as an example (Figure 4a), when kinematic waves move fast, non‐uniformity at a facet would disappear after growth. Accordingly, structures with reduced symmetry or THHs would be formed depending on the seed size (Figure 3c, d). However, if the 2D nucleation dominates the growth process, atoms could deposit at any site because the surface 2D nucleation barriers at different sites could be overcome. Then the kinematic wave would start to propagate. In general, non‐uniformities inside the facet planes could propagate to any direction, while propagations at edges and corner sites are geometrically constrained (Figure 4d). Thus, protrusions at corners would be formed when 2D nucleation dominates (e.g., at high precursor supersaturation, Figure S13b), and then decompose to 3 branches along <100> directions (according to the PGDs under these conditions and the coherent growth, Figure 4b, e and S13a). In analogy to organic dendrimers, the Au cubes could be seen as dendrimer generation 0 (G0), while those with 3 branches at each tip can be considered Au cube G1 (Figure 4b). If the Au cubes G1 are used as seed in the same synthesis, Au cubes G2 with another 4 branches at each previous branch are grown (Figure 4f). Due to the crowded space, not all the 48 branches could be easily seen, but the trend of growing new branches at the old branches is clear (Figure 4d–g). If the PGDs are <111> directions, the protrusions would be flattened by kinematic waves to form a larger Au cube.

This analysis was also validated for other seeds to prepare dendrimers (Figure 4c, h–k). For Au NRs, the 24‐facet NRs have sites at corners connecting 1 upper facet and 2 side facets (Figure 4h). Therefore, we can expect the protrusion to grow at those sites (under dendrites growth conditions), pointing to the <100> directions (Figure 4i, Figure S14). Similarly, Au NR G2 could be obtained. The same phenomenon was observed when using larger Au NRs as G0 (Figure S15). When Au octahedra were used as seeds, the shape evolution to form THH‐like intermediates co‐occurred with the branching process, which could explain the complexity of the resulting products (Figure S16).

The further growth of cubes has been recently researched by in situ experiments, which supports the suitability of SBKT to describe and predict NP growth at PGDs (Figure S17).^[32]^ Notably, while this phenomenon has been previously termed the deposition‐diffusion growth mode or thermodynamically‐kinetically controlled growth,^[1]^ SBKT‐based analysis provides a more advanced and straightforward approach. In the deposition‐diffusion growth mode, the location of atom deposition, the driving force for atom diffusion, and the sites where it diffuses to, all need to be carefully investigated under the given conditions. The SBKT provides a more straightforward approach to answer these questions: 1) the atoms could deposit at any position on the nanocrystal (which is reasonable from the perspective of chemistry), but at a faster rate or a larger possibility on those terraces perpendicular to the PGDs (that is why these directions are called the PGDs); 2) the driving force for the atom diffusion can be understood by the concept of half‐crystal sites. For any atom located at the surface, there are two sets of bondings: one with the constitute atoms (i.e., interior and neighboring surface atoms) and the other with the environment (i.e., dangling bonds or interactions with the surrounding solution). The former would make the atom stay, and the latter would make the atom detach. Atoms with stronger interactions with the surrounding medium would tend to diffuse to sites with a greater coordination environment (i.e., where the interactions with the surface atoms are more favorable than with the surroundings). This movement is easier by surface diffusion than through a dissolution‐deposition mechanism since it does not need to break all bonds already formed with vicinal surface atoms. However, the amount of sites fulfilling these conditions is limited and cannot be easily created (as discussed in Figure 2c). For these reasons, this might not be the main pathway for surface diffusion. Another type of special site is the half‐crystal site, where the strength of the two sets of interactions is similar. The half‐crystal sites are typically the front of the layer and can be created by surface 2D nucleations. Thus, the atoms tending to leave could diffuse to these sites. This could be the main type of layer advancement mechanism. Another form of layer advancement could be the direct deposition of atoms from solution at the layer front (or half‐crystal sites). However, it is less likely to occur, as the amount of half‐crystal sites is small compared to the total number of surface sites). Thereby, it is possible to answer the questions raised by the deposition‐diffusion growth mode.

## Conclusion

In conclusion, we have developed a symmetry‐based kinematic theory, SBKT, to explain the shape evolution of nanocrystals. The symmetry of crystal lattice and seed morphology, together with the PGDs defined by the growth environment, can help predict the growth trajectory. Thereby, it allows rationalizing the morphologies adopted by crystalline NP during growth and the advancement of retrosynthetic analysis in the field of nanomaterials. Moreover, SBKT is demonstrated to be beneficial in explaining symmetry‐breaking processes in a simple manner. This fact includes the formation of Au NRs and the traditionally elusive Au dendrimers, as it is experimentally proven in this work. However, testing for broader applicability, the SBKT could also be used to explain literature‐reported results.

The key to the SBKT success relies on adopting symmetry concepts into a NP growth analysis based on the classic kinematic theory and by considering the properties of kinematic waves (especially the interactions at nanoscale) in an unprecedented manner. The physical foundation of the SBKT is that the crystal grows via a surface nucleation process and layer advancements. Although the mathematical foundation is Frank's kinematic wave theory, complicated mathematical calculation is no longer needed in SBKT analyses. By doing this, we make it applicable for practical usage, as SBKT can accommodate all conventional growth mechanism criteria, from the stability of facets and Wulff constructions to deposition‐diffusion growth mechanism, among others. Moreover, it could also reconcile the previously cataloged thermodynamically‐controlled and kinetically‐controlled growth concepts via the coherent growth mechanism. Hence, SBKT emerges as a versatile theory for understanding crystal growth, with significant advantages with respect to current crystal growth theories (as summarized in Table 1). An illustrative example is the shape evolution of spherical Au seeds (Figure S12) for which none of the theories in Table 1 could successfully predict growth morphologies. However, SBKT can anticipate the formation of octahedra, truncated cubes, or rods, which are morphologies that can be experimentally realized (Figure S12) depending on the experimental conditions.^[27]^

**Table 1:** A comparison between different crystal growth theories.

		SBKT		BCF theory		Classical kinematic theory		Wulff construction		Deposition‐diffusion mechanism		Stability of facets
Main content		Foundations: BCF theory and the classical kinematic theory; Further focusing on the PGDs and the properties of the kinematic waves at nanoscale		The growth is validated by 2D nucleation (or screw dislocation) and layer advancement		The collective behavior of the layer advancements		In equilibrium the distances of the crystal faces from a point within the crystal are proportional to the corresponding specific surface energies		Growth is proceeding by atom deposition at corners and their subsequent diffusion to facets		Facets of low energy remain and high energy facets disappear
Main inputs of the theory		PGDs		Degree of supersaturation, surface energies		Full slowness vector plots		Surface energies		Relative rates of atom deposition and diffusion		Surface energies
Source for input		Pre‐experiments, reports with similar growth conditions, etc.		Chemical handbooks and experience		Not possible in real case		Theoretical calculations		Pre‐experiments		Theoretical calculations
Mathematical rigor under corresponding conditions		Yes		Yes		Yes		Yes		No		Yes
Illustration of the growth trajectory		Yes		Only in the case of screw dislocation driven growth		In theory		No		In part		No
Shape prediction		Yes		Only in the case of screw dislocation driven growth		No		Only the equilibrium shape		In part		No
Shape prediction for growth of a spherical seed		Yes		No		No		No		No		No
Explanation of symmetry breaking process		Yes		No		No		No		In part (heterogeneous growth)		No

The hierarchy of the concepts in the SBKT is (level 1) shape evolution; (level 2) PGDs, kinematic wave; (level 3) surface nucleation, layer advancement; (level 4) atom deposition, surface atom diffusion. Atom deposition and surface diffusion are the fundamental growth processes, and are highly influenced by the growth environments such as precursor concentrations, type of surfactants, ligands, nuclei or seed structures, temperature, etc. These factors can greatly influence the surface energies and taking full consideration of all these factors is quite difficult when attempting understanding nanocrystal growth solely using thermodynamic concepts. However, this issue can be avoided with the SBKT, since it mainly focuses on concepts located at higher hierarchical levels, i.e., the PGDs and properties of kinematic waves, to illustrate the shape evolution. The growth trajectory would be defined by the PGDs, that could be accurately determined by pre‐experiments, and modified by the properties of kinematic waves under the corresponding growth conditions. Estimation of PGDs according to thermodynamic arguments (surface energies), the directions perpendicular to the surface with the highest energies are likely to be the PGDs, and only those surfaces lying on the symmetry element of mirror planes need to be considered. The properties of kinematic waves include propagation, dissipation, and interaction. Propagation is the main property, and we have illustrated it through the concept of the half‐crystal site. However, in practice, we mainly need to consider the dissipation and interaction properties. The dissipation would occur at edge and corner sites and lead to smooth exposed facets. Combining the PGDs (which could help to build the framework of shape according to the lattice symmetry, Figure 3a) and the dissipation behavior of kinematic waves shall allow us to gain insights into shaped NP growth. Finally, the interaction between kinematic waves could lead to coherent growth, which might hold the key to the symmetry breaking processes.

Overall, the SBKT could be potentially used for the rational synthesis of desired NP morphologies, including complex ones such as dendrites. In addition, it should be possible to extend its use to macroscopic systems, thereby opening access to crystal design for multiple disciplines. Besides the clear understanding of the morphogenesis, SBKT also has excellent prospects to guide the retrosynthesis of discrete NPs and to identify possible transitional species. In the design of nanomorphologies, some post‐growth strategies, such as Ostwald ripening and structure conformation changes (like self‐coiling of nanowires^[33]^ or self‐folding of nanobelts[^34^, ^35^]), can be adopted together with the SBKT. The combination of growth and conformation changes has already been found to lead to complicated biomorphs.^[36]^ Furthermore, some particle‐mediated growth mechanisms have been found in recent years, such as oriented attachment^[37]^ and mesocrystal formation.^[38]^ These non‐classical processes can be combined with the SBKT to extend our full ability to design nano morphologies. Once the understanding of non‐classical processes has been perfected, it might be possible to achieve the total synthesis of inorganic materials like those of the total synthesis of natural organic products.

## Conflict of interest

The authors declare no conflict of interest.

1

## Supporting information

As a service to our authors and readers, this journal provides supporting information supplied by the authors. Such materials are peer reviewed and may be re‐organized for online delivery, but are not copy‐edited or typeset. Technical support issues arising from supporting information (other than missing files) should be addressed to the authors.

Supporting Information

## Data Availability

The data that support the findings of this study are available in the supplementary material of this article.
