# Supplementary material for: A Symmetry‐Based Kinematic Theory for Nanocrystal Morphology Design
Source: Angew Chem Int Ed Engl. 2022 Mar 14;61(20):e202200753. doi: 10.1002/anie.202200753 (PMC9310755; doi:10.1002/anie.202200753)
Supplement: Supplementary file 1 — Supporting Information [file ANIE-61-0-s001.pdf]

Angewandte Chemie
Eine Zeitschrift der Gesellschaft Deutscher Chemiker

Supporting Information

A Symmetry-Based Kinematic Theory for Nanocrystal Morphology Design

B. Ni, G. González-Rubio, F. Kirner, S. Zhang, H. Cölfen**

Chemicals: $HAuCl_4 \cdot 3H_2O$ (99.99 % (metals basis)), was purchased from Alfa Aesar. $AgNO_3$ (>99.9 %) was purchased from Roth. Hexadecyltrimethylammonium bromide (CTAB, >99 %), $NaBH_4$ (>98 %) and ascorbic acid (AA) were purchased from Acros Organics. Hexadecyltrimetryammonium chloride (CTAC, >98.0 %) and 1-decanol (>98 %) were purchased from Sigma-Aldrich. HCl solution (37%) was purchased from VWR. All reagents were used as received without further purification.

Synthesis of Au nanorods (NRs): The procedure was adopted from the literature[1]. In general, 1-2 nm Au seeds were synthesized in the first step; then small Au NRs (13.9 nm in length and 4.5 nm in width) were synthesized based on the small Au seeds. The small Au NRs were used as seeds in the following reactions. First, the concentration of the seeds (small Au NRs) was adjusted according to the optical absorption at 400 nm in the UV-vis spectrum, which was tuned to be 10 (optical path length of 1 cm, corresponding to a concentration of ~4mM). In a standard procedure to synthesize larger Au NRs, 60 µL of $AgNO_3$ (0.01 M) and 20 µL of $HAuCl_4$ (0.05 M) were added into 2 mL of CTAB/decanol solution (CTAB: 50 mM, decanol: 11 mM) in a PE tube, and sonicated to get a transparent solution. Then 16 µL of AA (0.1 M) was added into the solution. In the following steps, 10 µL of seeds and 50 µL of HCl (1M) were injected into the solution. The PE tube was vigorously shaken and quickly put into a water bath at 35 ^{o}C. The size of larger NRs could be tuned by the amount of seed solution and HCl used in the synthesis (see corresponding relations at Figure S2-6). As for the synthesis for symmetry breaking studies (Fig 3c, 3d, S8), only the seed type has been changed in the last step. The seed concentration and other relevant conditions remained the same.

Synthesis of Au dendrimers: Different Au nanoparticles (NPs) were used as seeds here, their concentrations were adjusted according to the optical adsorption at 400 nm, which was tuned to be 10 (optical path of length 1 cm). In a typical synthesis, 30 µL of $AgNO_3$ (0.01M) and 10 µL of $HAuCl_4$ (0.05M) were added into 2 mL of CTAB/decanol solution (CTAB:50 mM, decanol:11 mM) in a PE tube, and sonicated to get a transparent solution. Then 160 µL of AA (0.5 M) was added into the solution. In the

following steps, 10 µL of seeds and 20 µL of HCl (1M) were injected into the solution. Then the PE tube was vigorously shaken and quickly put into a water bath at 16°C. The generation of dendrimers could be tuned by the amount of seed solution. Higher Generation could be obtained with less amount of seeds. On the other hand, G2 dendrimers could also be fabricated by using G1 dendrimers as seeds.

Characterizations: The morphology and size of the products were determined by a Zeiss Libra120 transmission electron microscope (TEM) at 120 kV, Zeiss Gemini 500 scanning electron microscope (SEM). A JEOL 2200FS HRTEM operated at 200 kV, was used to perform high-angle annular-dark-field (HAADF) scanning TEM (STEM). Atomic resolution HAADF/ABF-STEM images were acquired using a probe-corrected Titan Themis microscope operated at 300 kV. A Cary 50 UV-Visible Spectrophotometer from Agilent Technologies was used for UV-Vis spectrum characterizations. The illustration polyhedra used in the paper were created by the WinXMorph software developed by Werner Kaminsky[2].

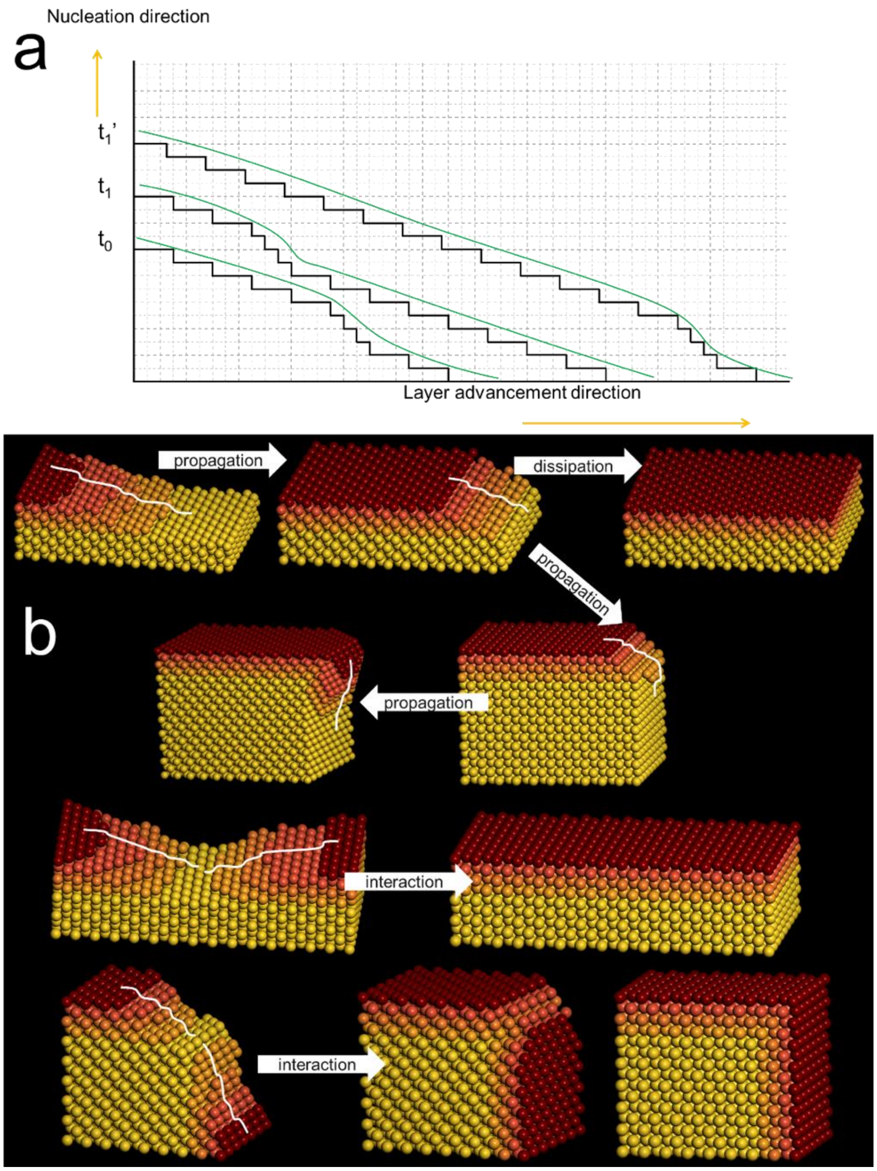

Figure S1. The movement, interactions, and dissipation of kinematic waves

(a) Green lines indicate the outline of the shape. If the one-atom steps move slower than the multi-atom steps, the front step can leave the non-uniformity, and the back step can be caught by the latter multi-atom steps. Accordingly, the non-uniformity would move from the center (t_0) to the left (t_1).

Similarly, if the one-atom steps move faster than the multi-atom steps, the non-uniformity would move from the center (t_0) to the right (t_1').

The kinematic wave is not really a wavy structure. It is the collective movement behavior of the layer advancement. The mathematics of the movements of such a non-uniformity can be described by waves. A detailed analysis is not necessary here. The method was first used in the road traffic systems, developed by Lighthill and Whitham at 1955[3]. Frank then introduced this method into the growth of crystals[4-5]. He also created a polar diagram of the slowness vectors to illustrate shape evolution. However, the connection between kinematic waves and the polar diagram was week. The polar diagram concerns all the growth rates of the possible facets of one particle. Thus, it’s hard to apply to real systems.

(b) White lines indicate the terraces, which might be non-uniformities in the structures. The movement of the white lines can be regarded as the kinematic waves.

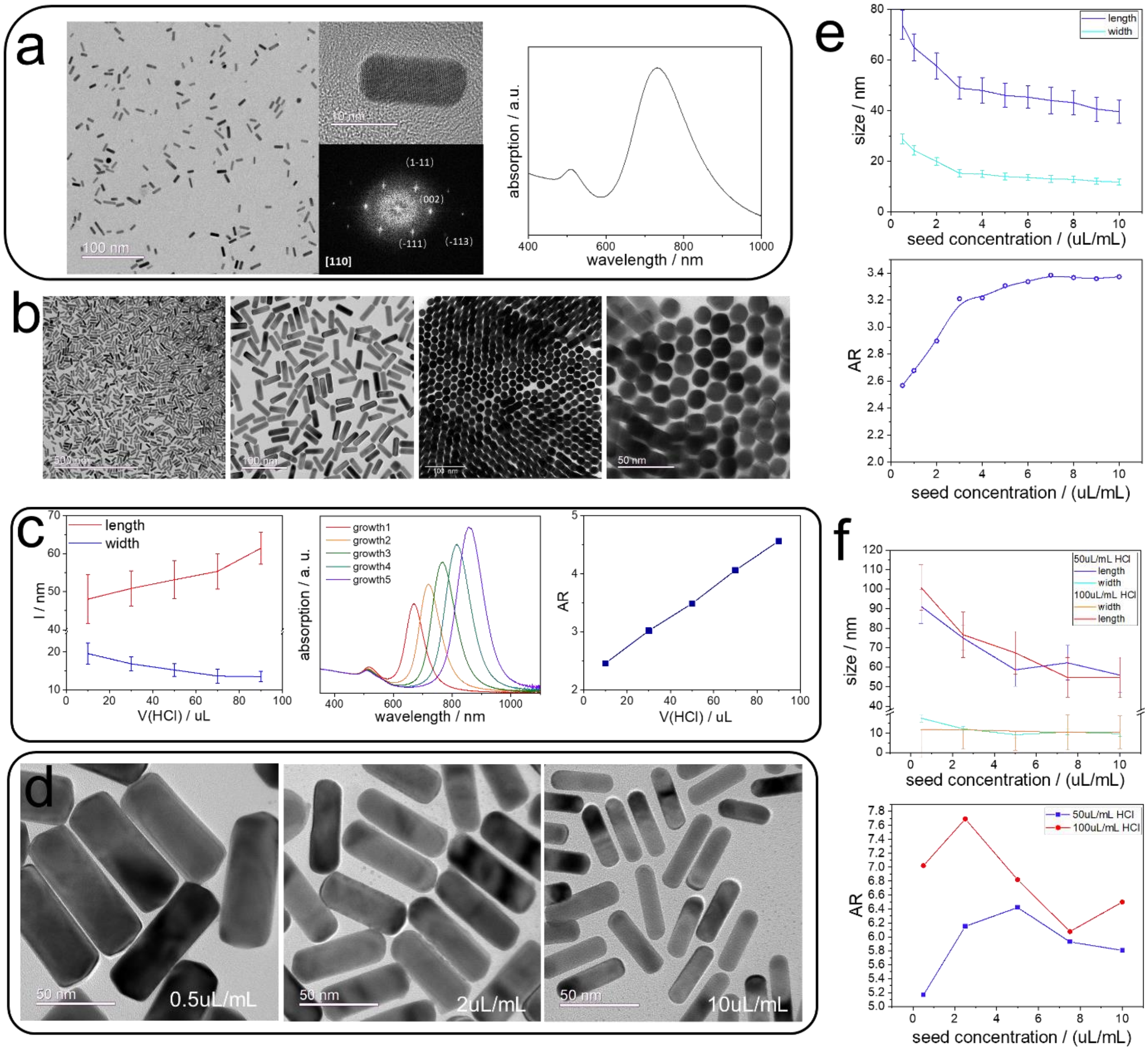

Figure S2. Au NRs of high quality are produced in a 3-step synthesis.

(a) Small Au NRs used as seeds to grow larger NRs were investigated by TEM and UV-Vis spectroscopy. The small Au NRs were 13.9 nm in length and 4.5 nm in diameter.

(b, c) The size and aspect ratio (AR) of Au NRs can be tuned by the amount of HCl solution. The depicted TEM images are the results of the growth3 under standard synthesis conditions.

(d) The size and AR of the NRs can be tuned by varying the seed concentration. The total amount of HCl used in this synthesis was 50 µL. The concentrations of seeds from left to the right were 0.5 µL/mL, 2 µL/mL, and 10 µL/mL, respectively.

(e) Size and AR related to the concentration of seeds at a fixed HCl concentration of 25 µL/mL.

(f) Size and AR related to the concentration of seeds at a fixed HCl concentration of 50 µL/mL and 100 µL/mL.

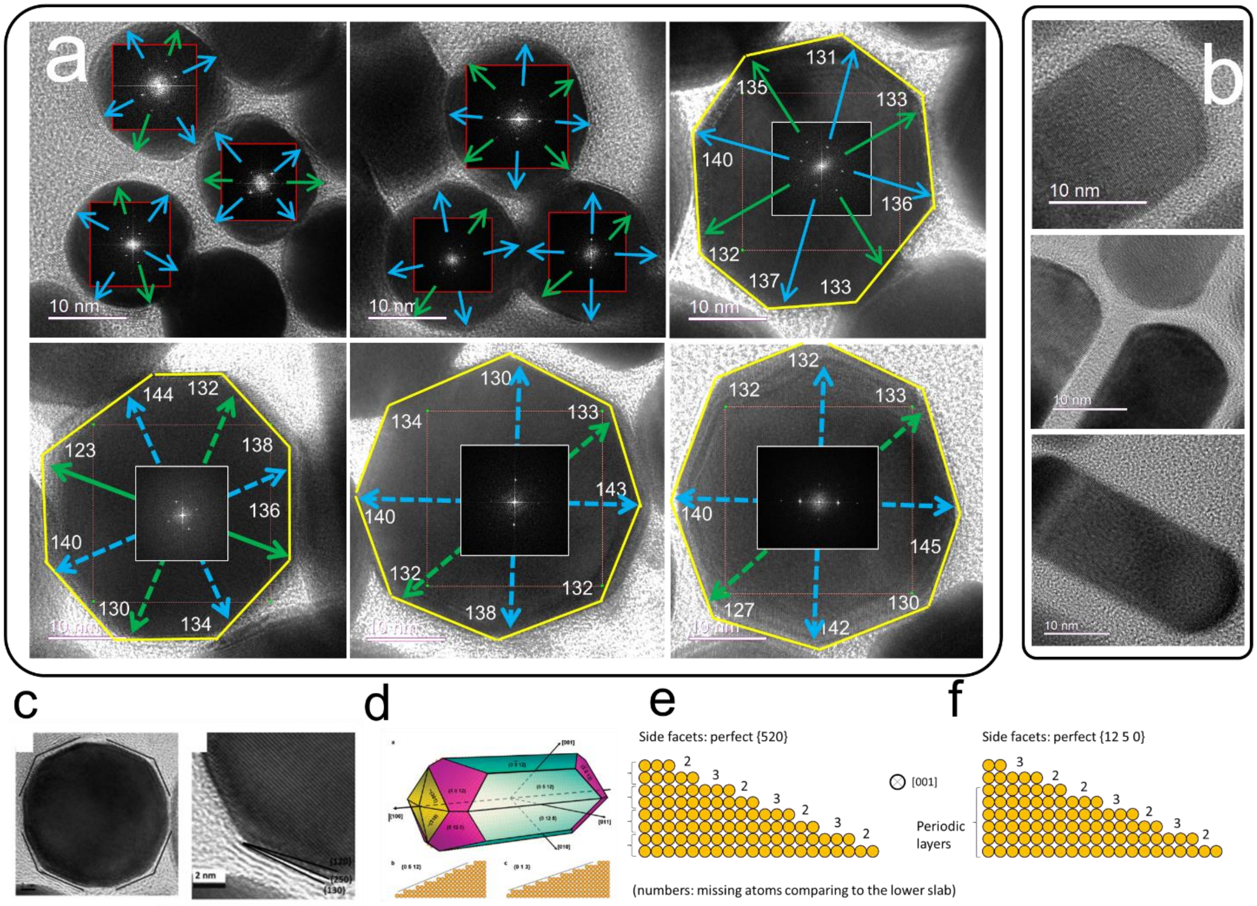

Figure S3. The structure of Au NRs is resolved by means of HRTEM.

(a) HRTEM images of the standing Au NRs of different sizes are depicted here.The patterns inside the red or white squares correspond to FFT patterns of the areas highlighted with red squares. The blue arrows indicate the <100> directions, while the green arrows denote the <110> directions. The yellow lines around the larger Au NRs sketch the side structure of the Au NRs, and the numbers are the angles between different side facets. The symmetry of the octagonal cross-section would decrease for an increasing diameter. Since the cross-sections of the NRs were not perfect, it was hard to explicitly determine the index of the exposed facets. Thus, explaining the growth mechanism from the perspective of exposed facets would encounter inevitable troubles. Our SBKT could avoid this problem just by considering the PGDs.

(b) HRTEM images of the NR tips show that the rod is along <100> directions

(c) The side facets of Au NRs are {5 2 0} facets. Adapted with permission from Ref. [6]. Copyright 2010, WILEY-VCH (Angew. Chem. 2010, 122, 9587 –9590)

(d) The side facets of Au NRs are {12 5 0} facets. Adapted with permission from Ref. [7]. Copyright 2011, ACS (Nano Lett. 2011, 11, 273–278)

(e, f) The atomic arrangement of the {5 2 0} facets and {12 5 0} facets looking along [001] direction.The difference between {5 2 0} facets and {12 5 0} facets is the frequency of the 2-atom steps and 3-atom steps.

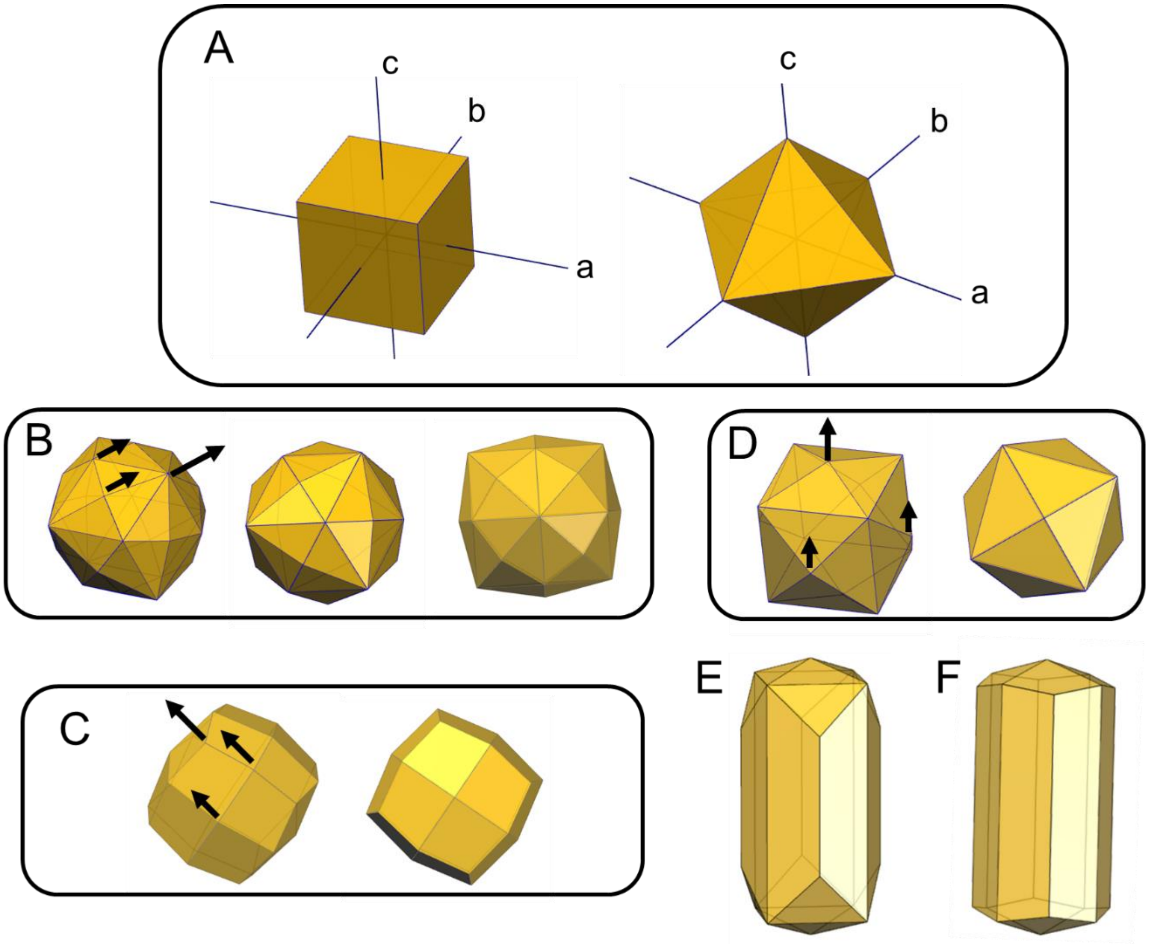

Figure S4. Schematic illustration of the <100> preferential growth directions.

(A) Illustration of the crystal directions in fcc lattice. There are 6 and 8 equivalent <100> and <111> directions in the fcc lattices, respectively, while there are 12 equivalent <110> directions in the fcc lattice. The corners of the cube are all pointing to the <111> directions, and the corners of the octahedron are pointing to the <100> directions. The centers of the cube or octahedron edges are pointing towards the <110> directions. If the <110> directions were the preferential growth directions (PGDs), and the high-index facets remain after the growth, sharp tips pointing to all the <110> directions should appear during growth. Thus, a hexoctahedron (B), which is enclosed by {hkl} facets (here {321}), or a trapezohedron (C), which is enclosed by {hkk} facets (here {511} facets) should be the possible shapes. The afore constructed morphologies maintain the $\frac{4}{m}\bar{3}\frac{2}{\mathrm{m}}$ symmetry. If we stretch the particles in <110> directions to create a nanorod (along with the black arrows, for example), new facets would appear, which contradicts the assumption of an iso-faceted structure. Furthermore, since the exposed facets are close to (1+√2 1 0) facets, such as {5 2 0} or {12 5 0} facets, which contradicts the afore constructions. Thus, the PGDs along <110> directions could be ruled out.

(D) Similarly, if <100> directions are the PGDs, tetrahexahedra (THH) enclosed by {hk0} should develop (here {520} facets), and if the THH is stretched in <100> directions, a nanorod (E) purely capped by {hk0} can be obtained without creating new facets (just elongating the edges at “side” facets), which coincide well with the experimental results (Figure S4). The driving force of the stretching (symmetry breaking) will be discussed in the following sections (Figure S6, 7). Here we obtained the PGDs simply according to the geometry of the particles, without the need to know the stabilization effects of Ag^+, CTA^+, and Br^- on different facets. The <100> preferential growth directions were further confirmed by using cubes as seed particles in the growth. (Figure S7). Based on the <100> PGDs, a 24-facet Au NR (E) and a 16-facet Au NR (F) can be constructed. The exposed facets are all the same (here {520} facets were used for illustrations). The evolution from E to F is discussed in Figure S7.

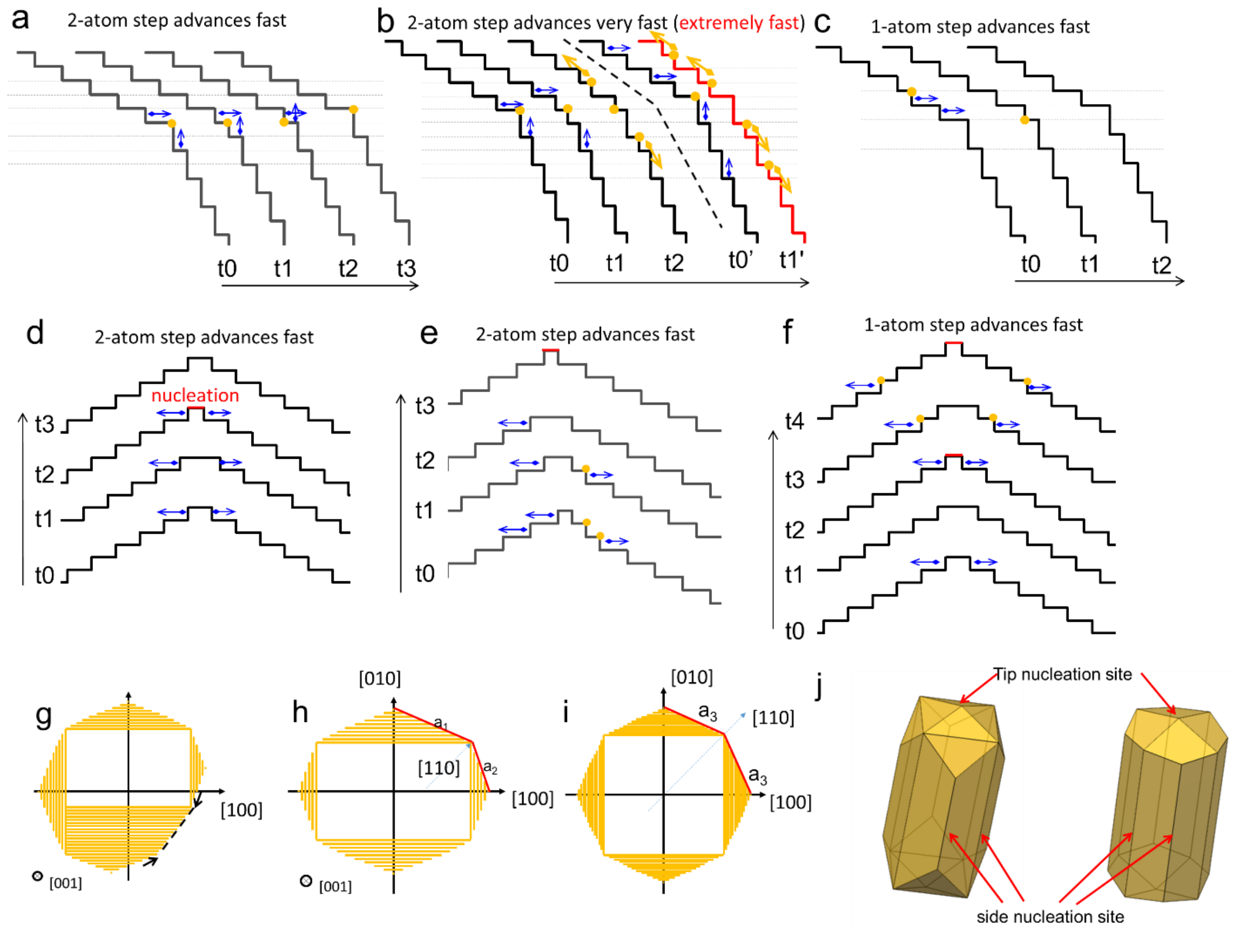

Figure S5 The movement, interactions and dissipation of the kinematic wave in the growth environments of the NRs here (see detail in **Synthesis of Au NRs**: $AgNO_3$ (0.01 M) + $HAuCl_4$ (0.05 M) + 2 mL CTAB/decanol solution (CTAB: 50 mM, decanol: 11 mM) + AA (0.1 M)).

(a-f) Different situations of the movement of steps.

Here, a 2-atom step is used to represent the multi-atom step (2-atom step or 3-atom step). The principles of movement and interaction are the same for 2-atom steps, 3-atom steps, or uneven steps. The illustrations show in a qualitative mode that the non-uniformities (1-atom steps) are created, move to different edges, and vanish, since “fast growth” is not equal to that only one kind of step moves while other kinds of steps are frozen.

(a) The steps meeting at the edges pointing to the <110> directions (named as <110> edges hereafter) would disappear and result in the enlargement of the Au NRs.

(b) If the 2-atom step moves much faster than the single-atom step, the single-atom step would move to the <100> edge.

(c) If only one non-uniformity propagates to the <110> edge, it will disappear, and the lower facet would move up by 1 step.

(d) A new terrace can be produced at the <100> edges, and the new nucleation process would advance the terrace, resulting in the enlargement of the NRs.

(e) If the 2-atom step moves much faster than the single-atom step, and only one non-uniformity reached the <100> edge (from the right side, for example), the center of the edge would move to the other side (to the left here).

(f) When the one-atom step moves much faster than the 2-atom step, non-uniformities would be created and propagate to the <110> edges, and then disappear there according to (d).

(g-i) An ill-defined octagonal cross-sections can evolve to a symmetrical octagonal shape.

(g) The non-uniformity cannot exist at the <100> edges since any non-uniformity there would rapidly vanish by new surface nucleation events (e-g). If there is a non-uniformity at the <110> edges (h), it would gradually disappear by the layer advancement according to the analysis at (b-d).

(h) Seemingly, there is no non-uniformity in this structure. However, the non-uniformity could be created at the <100> or <110> edges according to (b-g), and such non-uniformity could easily propagate to the adjacent edges in the form of kinematic waves. Since here $a_1 \neq a_2$, the created non-uniformity would reach the adjacent edges at different times, leading to the movement of edges. Thus, this structure is not stable.

(i) The only stable form of cross-sections is depicted here and can be described as octagonal.

(j) Possible nucleation sites of rods are pointed out in this subfigure. This should clarify explanations for aspect ratio (AR) tuning during growth.

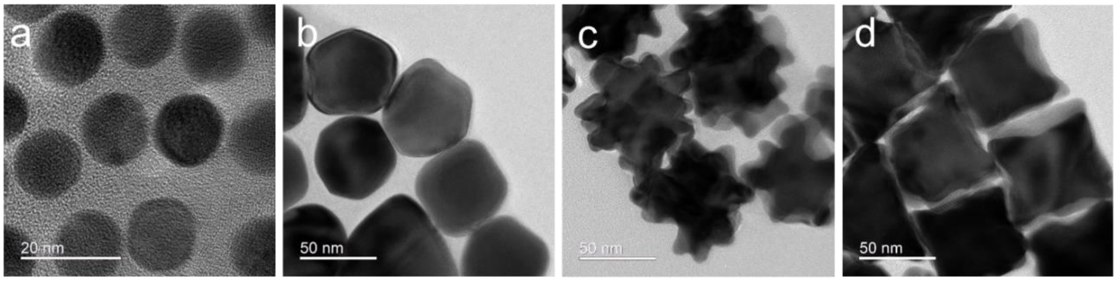

T (°)	Surfactant (mM)	AA (mM)	Typical TEM	FFT	PGD	T (°)	Surfactant (mM)	AA (mM)	Typical TEM	FFT	PGD
17	50 (B)	0.8			<100>	35	50 (B)	0.8			<100>
	50 (B)	40			<100>		50 (B)	40			<100>
	5 (B)	0.8			<100>		5 (B)	0.8			<100>
	5 (B)	40			<100>		5 (B)	40			<100>
							50 (CTAB)	0.8			Hard to identify
21	50 (B)	0.8			<100>		50 (CTAB)	40			<111>
	50 (B)	40			<100>	45	50 (B)	0.8			<100>
	25 (B)	0.8			<100>		50 (B)	40			Hard to identify
	25 (B)	40			<100>		5 (B)	0.8			<100>
	5 (B)	0.8			<100>		5 (B)	40			Hard to identify
	5 (B)	40			Hard to identify		50 (CTAB)	0.8			<100>
	50 (CTAC)	0.8			<111>		50 (CTAB)	40			<111>

Figure S6. PGDs at different conditions.

(a) Spheres (~12.4nm) used as seeds for exploring PGDs at various conditions. The synthesis procedure was similar to that of Au NR prepared using small Au NRs as seeds: 60 µL of $AgNO_3$ (0.01 M) and 20 µL of $HAuCl_4$ (0.05 M) were added into 2 mL solutions of different surfactant (B is a solution with 50mM CTAB and 11mM

decanol) in a PE tube and sonicated to get a transparent solution. Then, different amounts of AA solution were added to the growth solution (the concentrations in the table indicate the final concentrations in the reactant). In the following steps, 10 µL of sphere seeds (~4mM) and 50 µL of HCl (1M) were injected into the solution. The PE tube was vigorously shaken and quickly put into a water bath at different temperatures (the amount of HCl does not have a strong influence on the PGDs, see Figure S2, as well as the amount of $AgNO_3$ (data not shown)).

(b-d) Most of the syntheses lead to similar structures. Here are some typical TEM images with lower magnification than the TEM images at the table.

The PGDs were determined by both the FFT patterns of the lattice, as well as the shape of the structures (see shape analysis at Figure S7b). The results showed that the PGDs stay stable in a wide range of reactant concentrations and temperatures in this reaction system. The reason might be that supersaturation and temperature have a similar influence on all the possible growth directions. Thus, the PGD would not be dramatically influenced when the conditions slightly changed. However, if the surfactant changed, the surface nucleation energy barriers would significantly change. Thus, the surfactant system has a strong influence on PGDs.

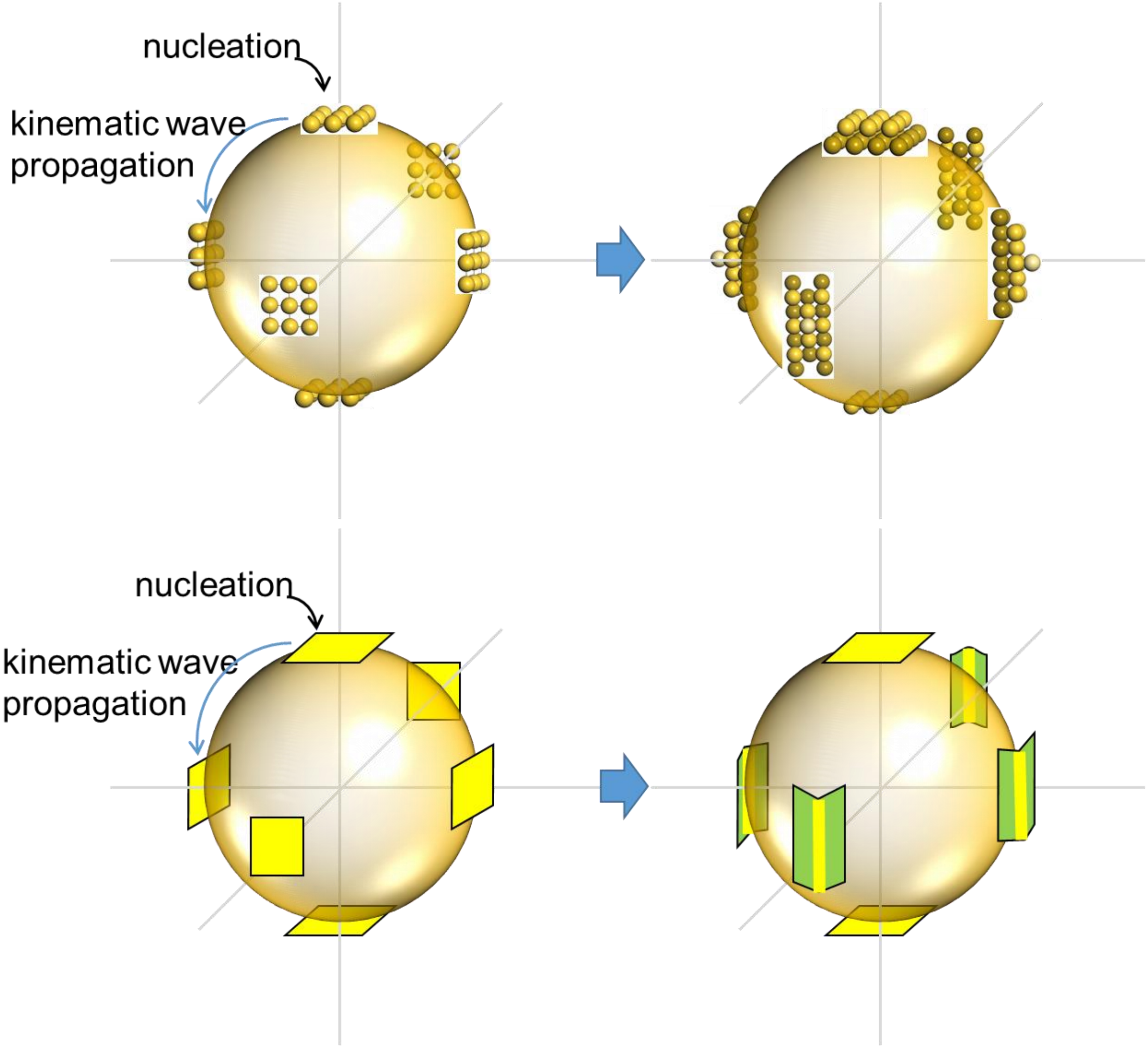

Figure S7. Concept of the coherent growth

Once a nucleation event occurs at top facets, the kinematic wave would start to propagate. If the kinematic wave can reach the adjacent nucleation sites before the next nucleation event occurs (the advent of a second kinematic wave), then this kinematic wave would change the structure of these sites, thereby diverging from that of the top and bottom nucleation sites. Meanwhile, if a new surface nucleation event occurs at this time (before the full dissipation of the first kinematic wave since it will eventually dissipate all non-uniformities), the possibilities of nucleation at the top/bottom sites and middle sites are different. Thus, the NP symmetry would be broken.

Spheres are used to indicate the high symmetry of interior particles, and only the nucleation sites are shown here for simplicity. The upper panel describes the atomic structures of the nucleation sites, while the lower panel illustrates the general situations of the nucleation sites (yellow planes indicate the nucleation sites). Thus, this model can be applied to spherical seeds, cubic seeds, octahedral seeds, etc.

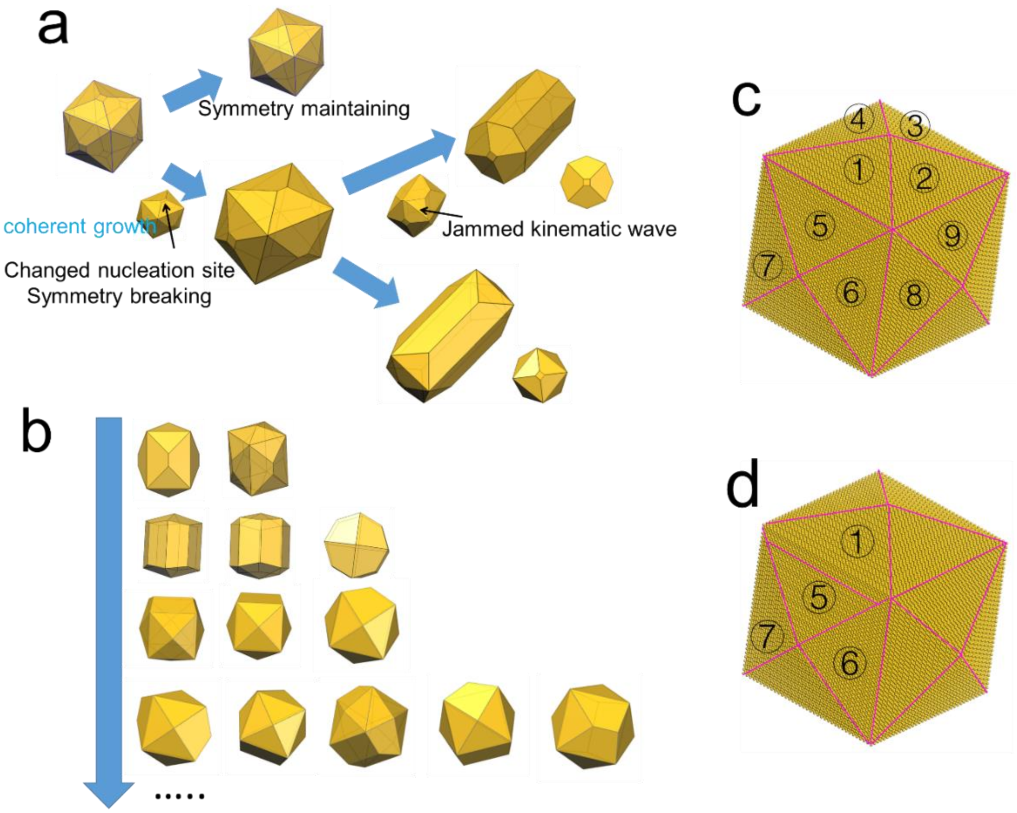

Figure S8. This figure sketches the symmetry-breaking process.

(a) Coherent growth to change the adjacent nucleation sites and the corresponding symmetry-breaking process
If there is no coherent growth, the symmetry would be maintained, and THH would be the only product. If the coherent growth occurs and changes the nucleation sites, the symmetry would be broken to form rods (24-facet Au NR). During the growth of rods, the kinematic wave could propagate from the tip to the side facets. If the kinematic wave is jammed at the junction of tip and side facets, the 24-facet Au NR will evolve to the 16-facet Au NR.

(b) The coherent growth is more dominant at high kinematic wave propagation rates. Thus, the symmetry would be further decreased. The pictures within the same row contain the same structure depicted from different angles. This figure might help to explain why an un-optimized synthesis experiment produces a series of nanoparticles that look quite dissimilar, even though they experienced similar or the same growth condition.

(c, d) An atomic model can help to understand the movement of the kinematic waves. Kinematic wave at side①can directly propagate to side②④⑤ but cannot directly move to side③⑦⑧. The propagation to side⑥⑦ can be intermediated by side⑤. If a huge kinematic wave (non-uniformity) is jammed at the edge between side① and ⑤, then the stuck non-uniformity connects side①⑥⑦. Accordingly, the kinematic wave can directly propagate from side① to side⑥⑦ and change the structure to a 16-facet one.

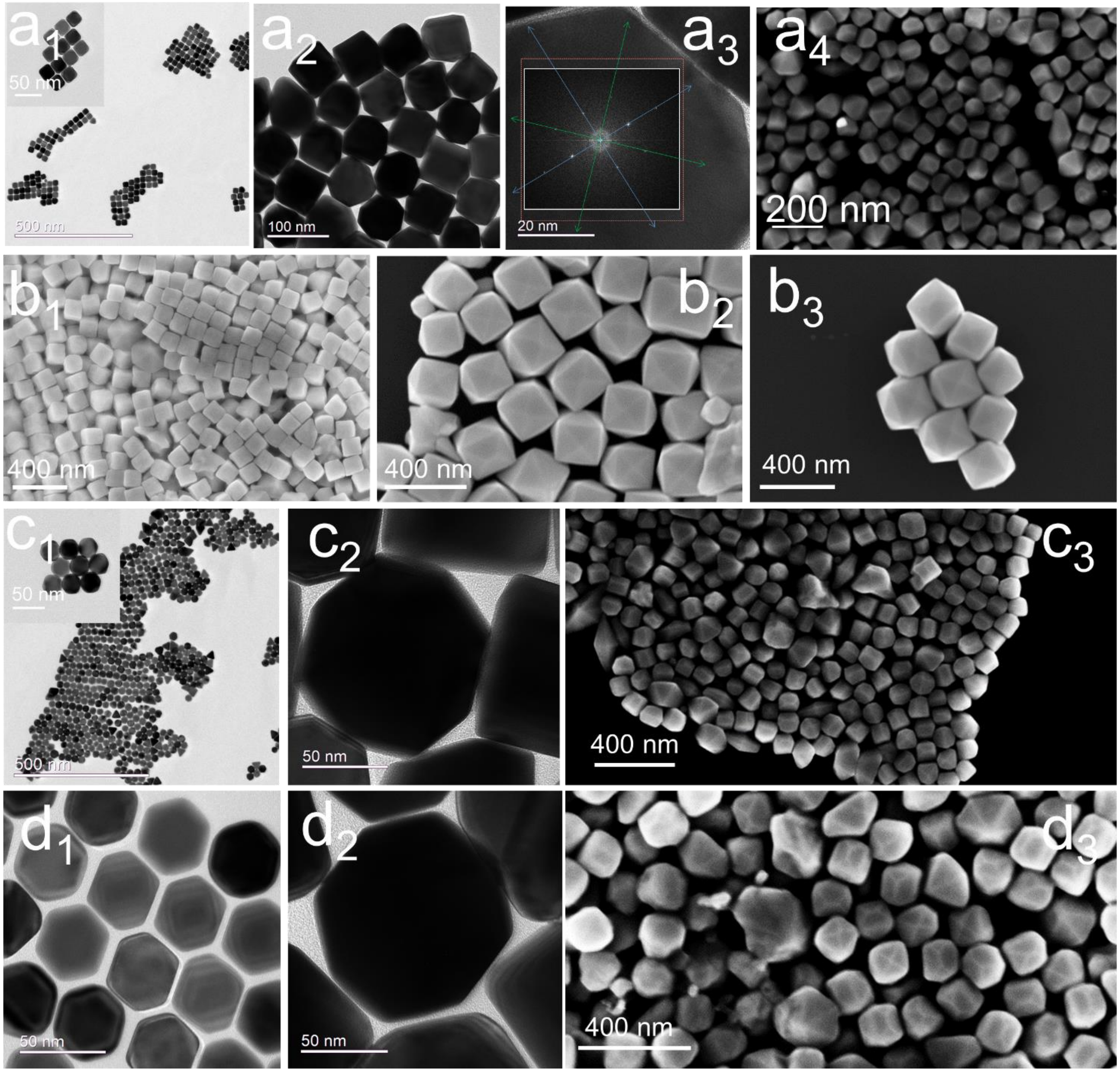

Figure S9. TEM and SEM images of particles grown from different seeds.

(a) Small Au nanocubes (a1) as seeds result in symmetry-broken THH like structure (a2-a4). a3 shows that the edges of the particles were pointing to the <100> and <110> directions, the same as the Au NRs.
(b) THH (b2,3) were obtained when larger Au nanocubes (b1) were used as seeds.
(c) Small Au octahedra (c1) as seeds lead to symmetry-broken THH like structures (c2-c3).
(d) When Au cuboctahedra (d1) were used as seeds, symmetry-broken THH like structure are obtained (d2-d3).

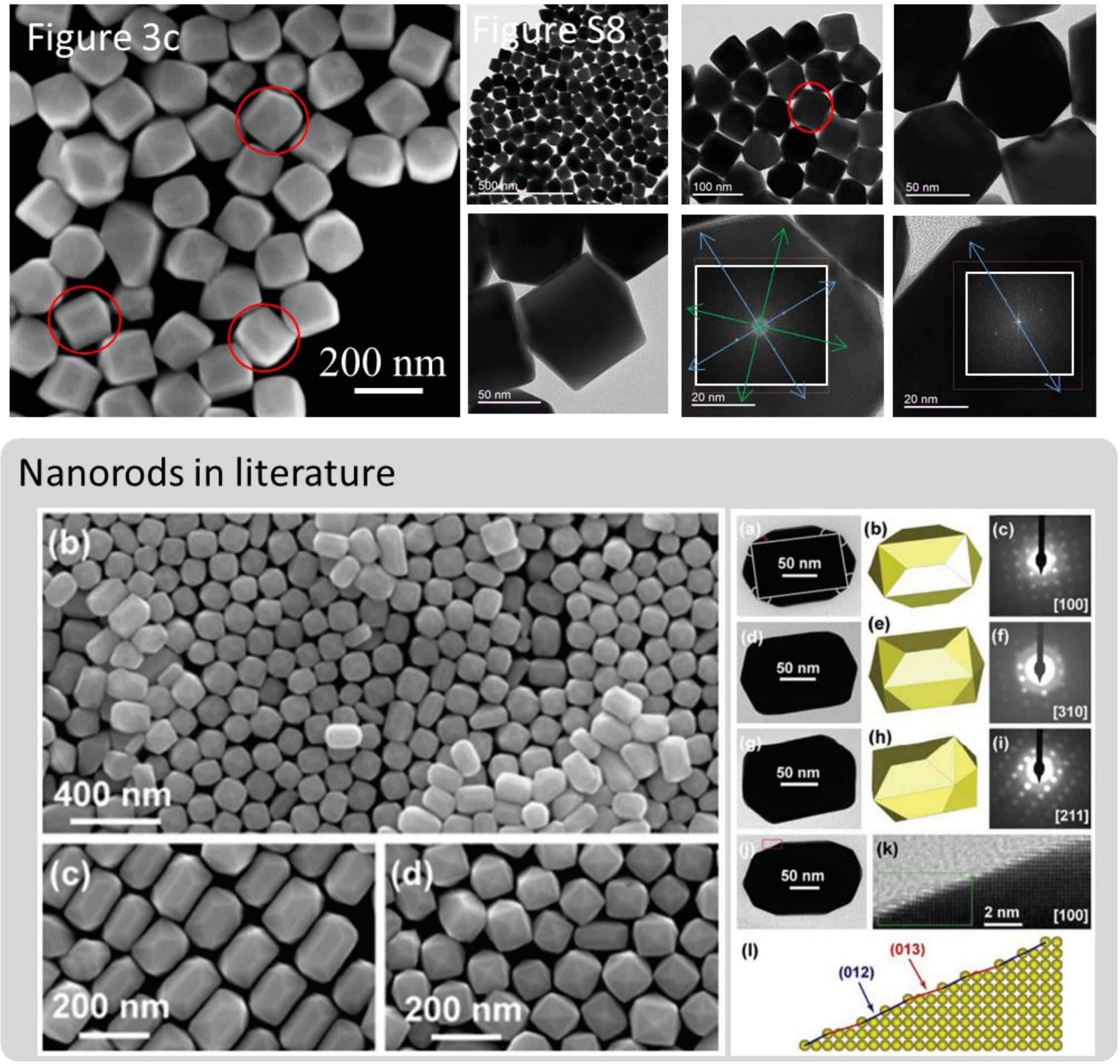

Figure S10. Comparison between our particles and Au nanorods reported in the literature. The particles inside the red circles have similar morphology with the reported results[8] (Adapted with permission, Copyright 2009, ACS), even though they have a higher aspect ratio.

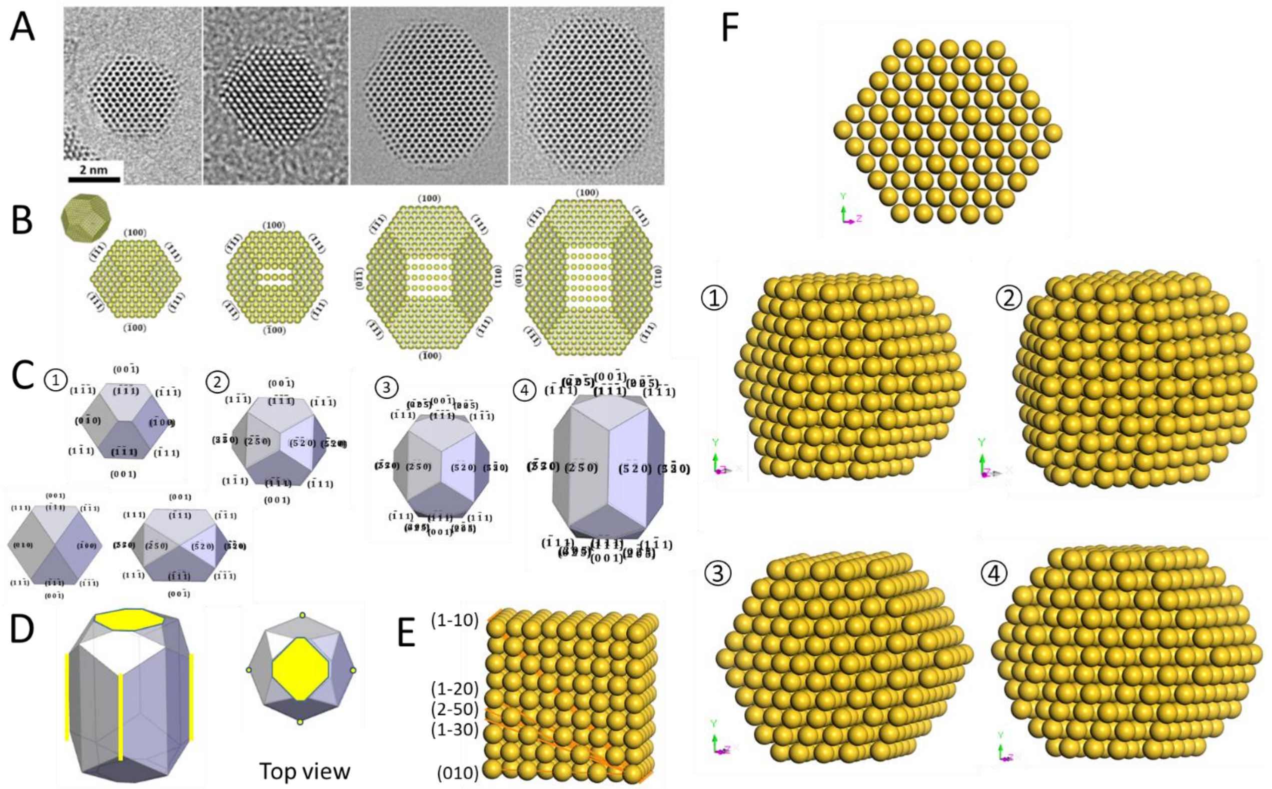

Figure S11. The symmetry breaking growth was previously researched by *in situ* TEM (A, B)[9] ((Adapted with permission, Copyright 2017, ACS)). However, the method could only provide 2D projection information of the process. Therefore, it cannot rule out the possibilities shown at (C). Many different structures could all have the same hexagonal projections (F) at the initial structure shown at (A)

(D) The yellow areas are the nucleation sites with the PGDs of <100> directions. (E) It can be found that (1-30), (2-50), and (1-10) facets could be the intermediate facets evolving from (010) facets to the (1-10) facets. Thus, the evolution shown at (C) should be very possible.

(F) Different structures having the same hexagonal projections. It is worth mentioning that the symmetry of (1,2,4) structures is already broken. The sizes of (100) facets at top/bottom and middle sites are different. However, this cannot be depicted by 2D projections. Furthermore, according to these atomic structures, the kinematic wave propagation from top/bottom (100) facets to the middle (100) facets is possible since they are closely connected.

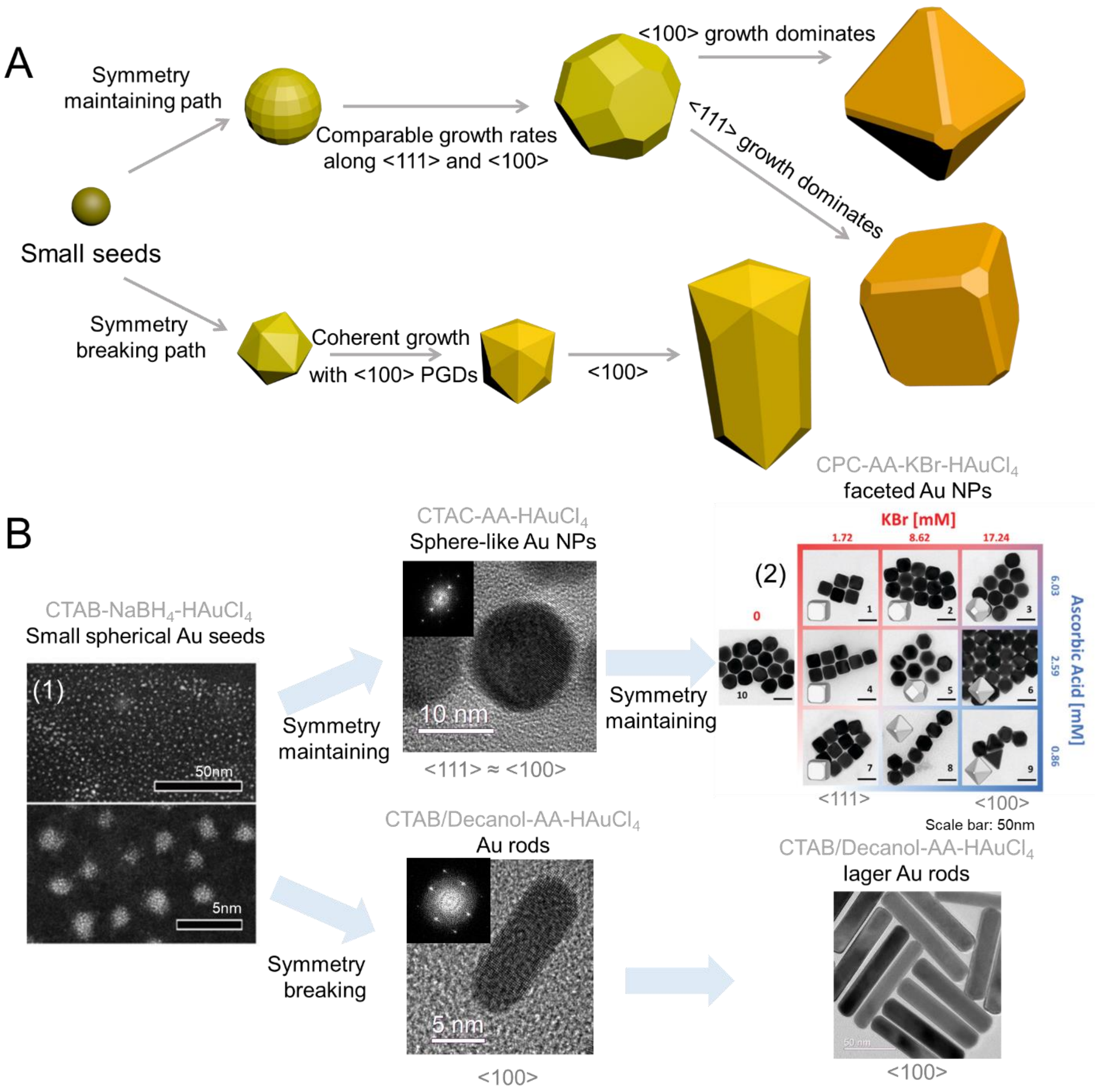

Figure S12. Sketch and experimental demonstration of the shape evolution

(A) scheme of the symmetry maintaining and symmetry breaking paths according to the SBKT.

(B) Experimental demonstration of the scheme. The vectors below the TEM images indicate the PGDs according to the crystallography. In the symmetry maintaining path, sphere-like particles could be formed if the growth rates along <111> and <100> are comparable. The shape cannot be adequately explained by the Wulff construction or the stabilization of certain facets since spheres show no preferred faceting. Moreover, the sphere-like particles could evolve to cubes or octahedra depending on the relative growth rates along corresponding directions, which could be experimentally obtained by slightly tuning the precursor concentrations (KBr and AA). It is worth mentioning that the concentration of KBr and AA here were much larger than the total amount of particle surface atom, and the changes in concentrations could still greatly influence the shapes. To our knowledge, the precursor concentrations are hard to be included in the theoretical calculations of surface energies, let alone the relationship between surface energy changes and the precursor concentration changes. However, the SBKT could avoid such challenges by taking advantage of pre-experiments to determine the

relative growth rates along different directions. The SBKT could also explain the symmetry-breaking process by considering the coherent growth mechanism, which is also a challenging task for other theories.

(1) Adapted with permission from Ref. [1]. Copyright 2019, ACS (ACS Nano 2019, 13, 4424−4435)
(2) Adapted with permission from Ref. [10]. Copyright 2020, RSC (J. Mater. Chem. C, 2020, 8, 10844)

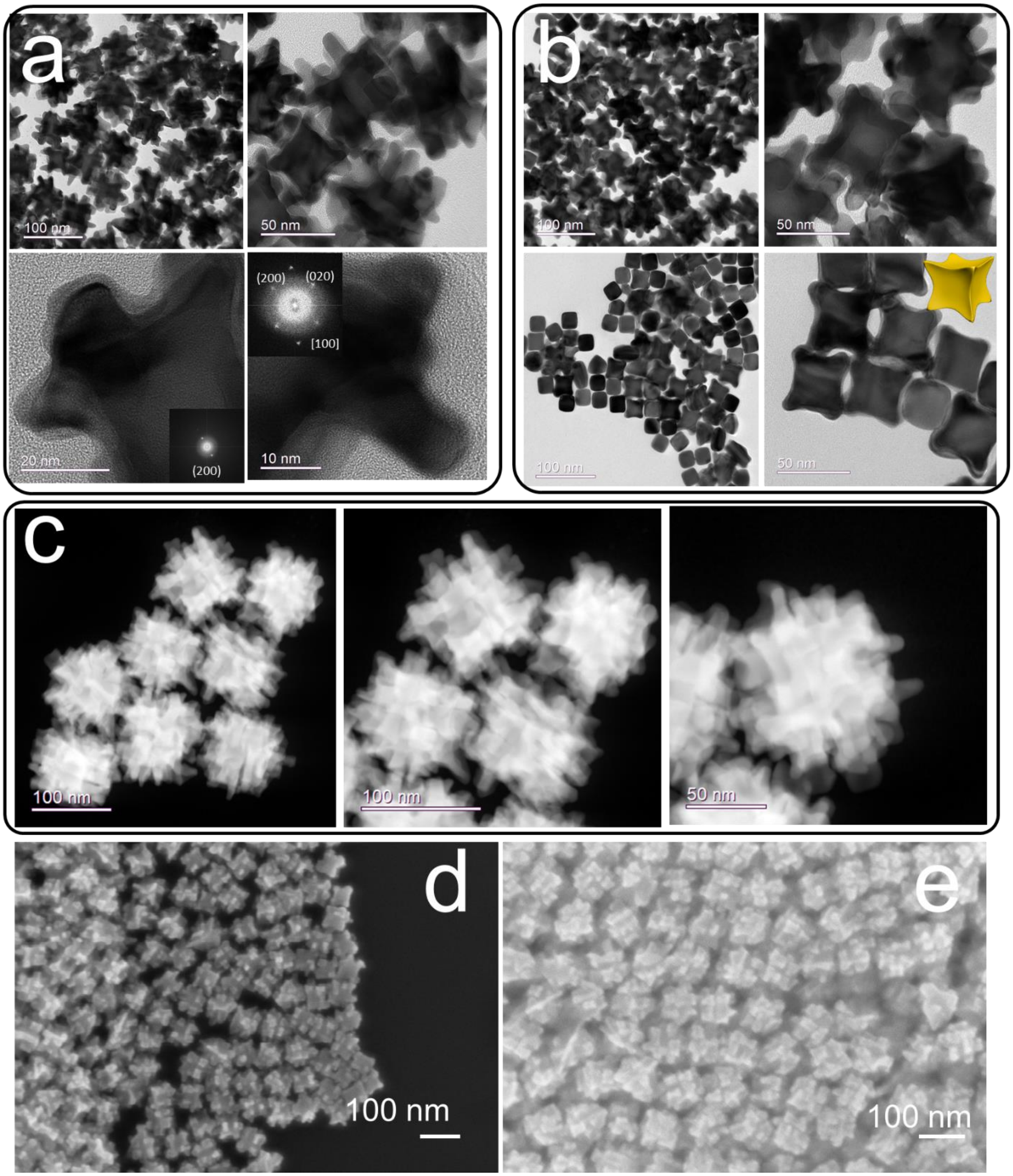

Figure S13. Using Au cubes as G0 to prepare dendrimers.

The cubes used here are the same as Figure S9a1.
When Au cubes were used as seeds, atoms would accumulate at the corners and eventually evolve to 3 branches (G1 structures, a). The intermediates from Au cube G0 to G1 agree well with the analysis (b, in this synthesis, half-amount of AA was used. The corners of the cube are producing protrusions, which indicate that the corner sites could accumulate atoms). Similarly, Au cube G2 has been fabricated by using G1 as seeds (c).(d, e) SEM images of the G1 and G2 structures, respectively.

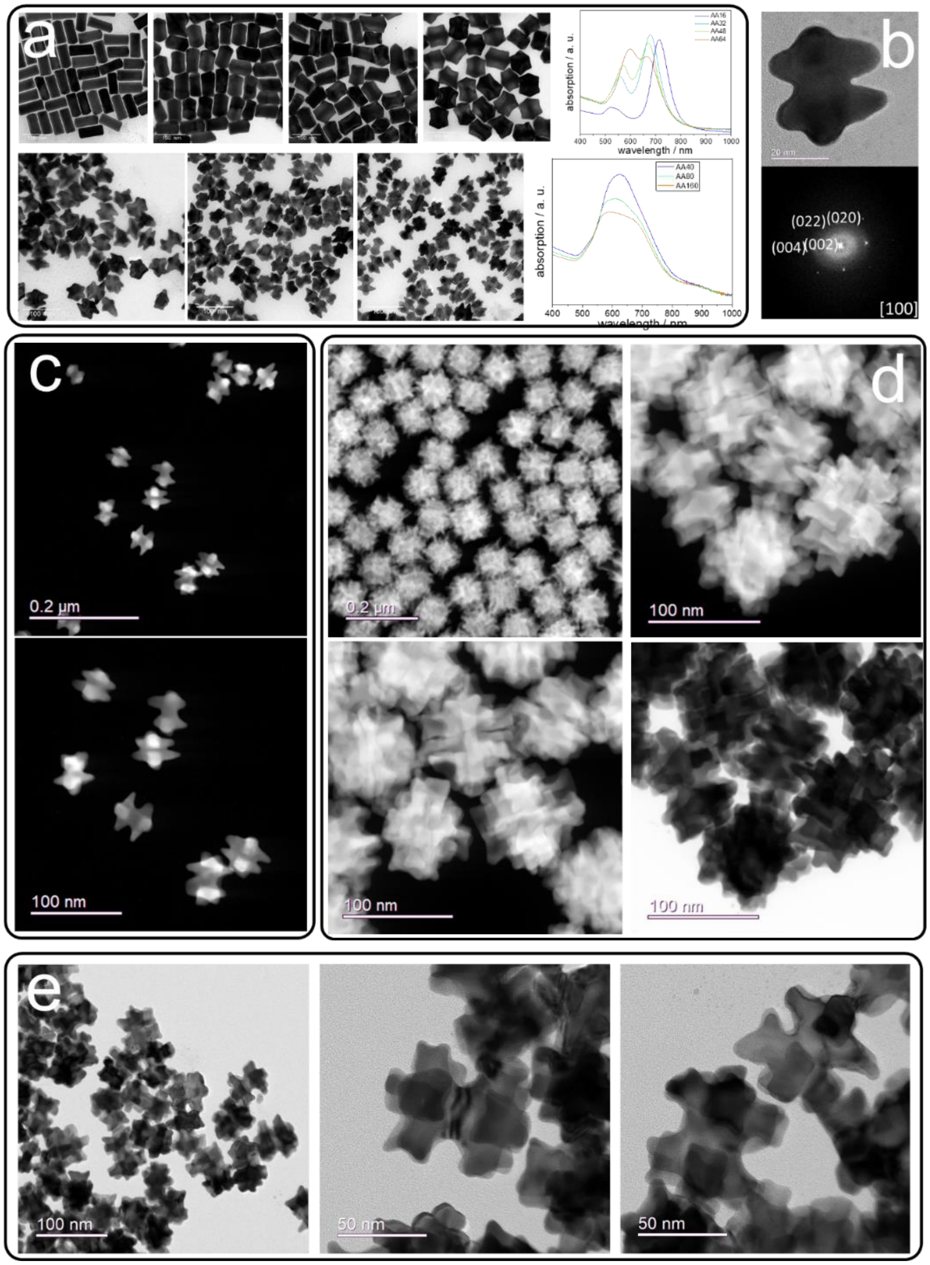

Figure S14. The dendritic growth of Au NRs was investigated by means of TEM, STEM, and UV/Vis spectroscopy.

(a) The shape of NRs would change when the amount of AA is increased in the synthesis of large Au NRs. The AA (0.1M) used in the synthesis was 16 µL, 32 µL, 48 µL, 64 µL from left to right in the first row, respectively. The AA (0.5M) used in the synthesis was 40 µL, 80 µL, 160 µL, and 64 µL from left to right in the second row, respectively.
When the amount of AA increases, which accounts for a faster reduction and higher supersaturation, branched structures would gradually evolve.

(b) HRTEM image of the Au NR G1 dendrimer.
The corresponding FFT patterns indicate that the branching occurs in <100> directions, which are the PGDs in the synthesis.

(c, d) STEM images of the Au NR G1 dendrimer (c) and G2 dendrimer (d).

(e) TEM images showing the branching tips of the Au NR G2 dendrimer.

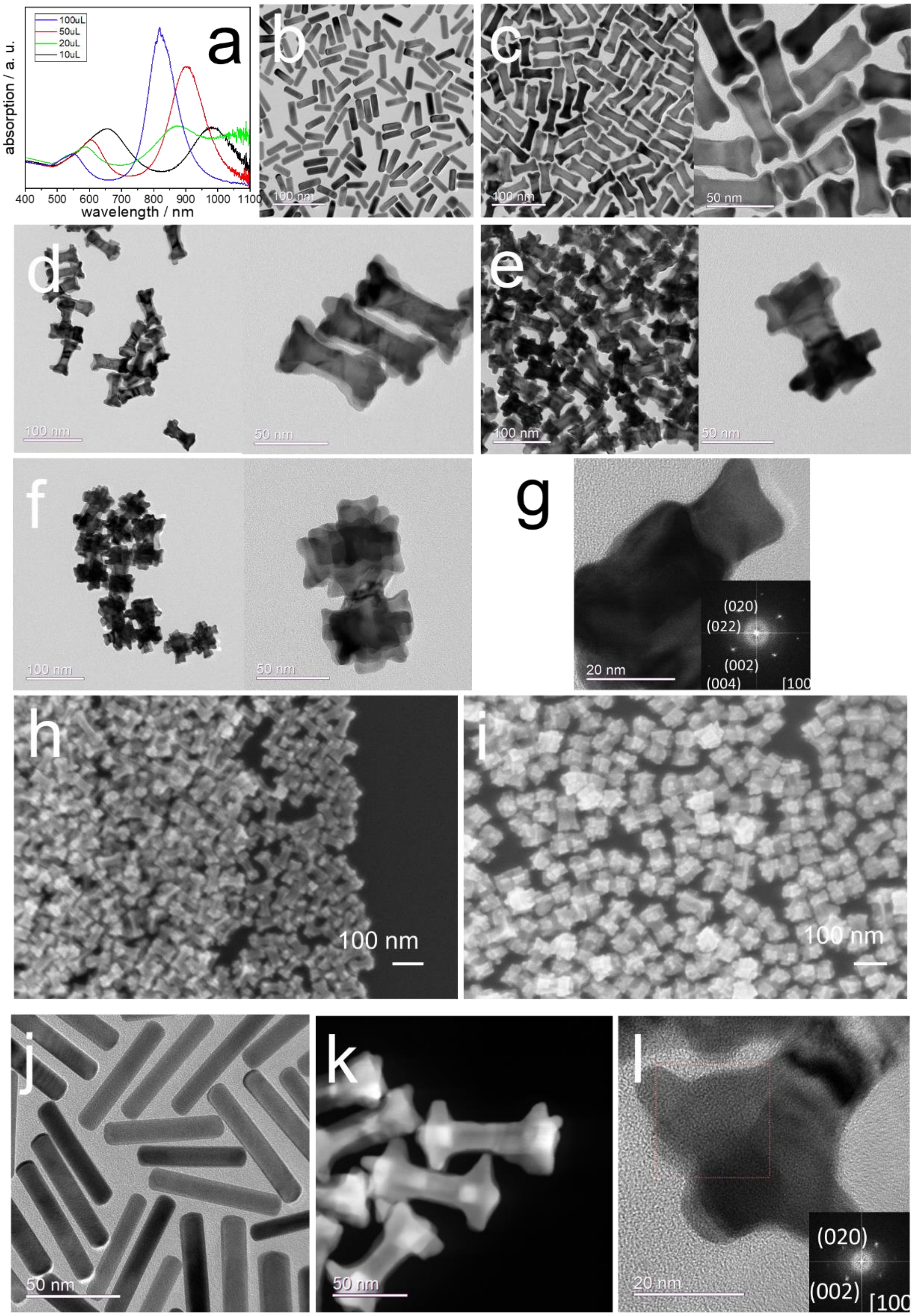

Figure S15. Larger Au NRs were used as G0 to prepare dendrimers.

Tuning the concentration of seeds could lead to different generations. The reason might be due to the consumption of precursors during the synthesis. The TEM images show that the growth of the branches is fractal.

(a) UV-Vis spectra of dendrimers depicted in c-f. The legend in the figure indicates the amount of seeds used in the synthesis.
(b) original Au NRs as seeds (G0).
(c) 100 µL seeds were used in the synthesis; (d) 50 µL seeds were used in the synthesis; (e, g) 20 µL seeds were used in the synthesis; (f) 10 µL seeds were used in the synthesis.
(h, i) SEM images of the G1 (d) and G2 (f)
(j-l) Another size of Au NRs as G0 to prepare dendrimers.

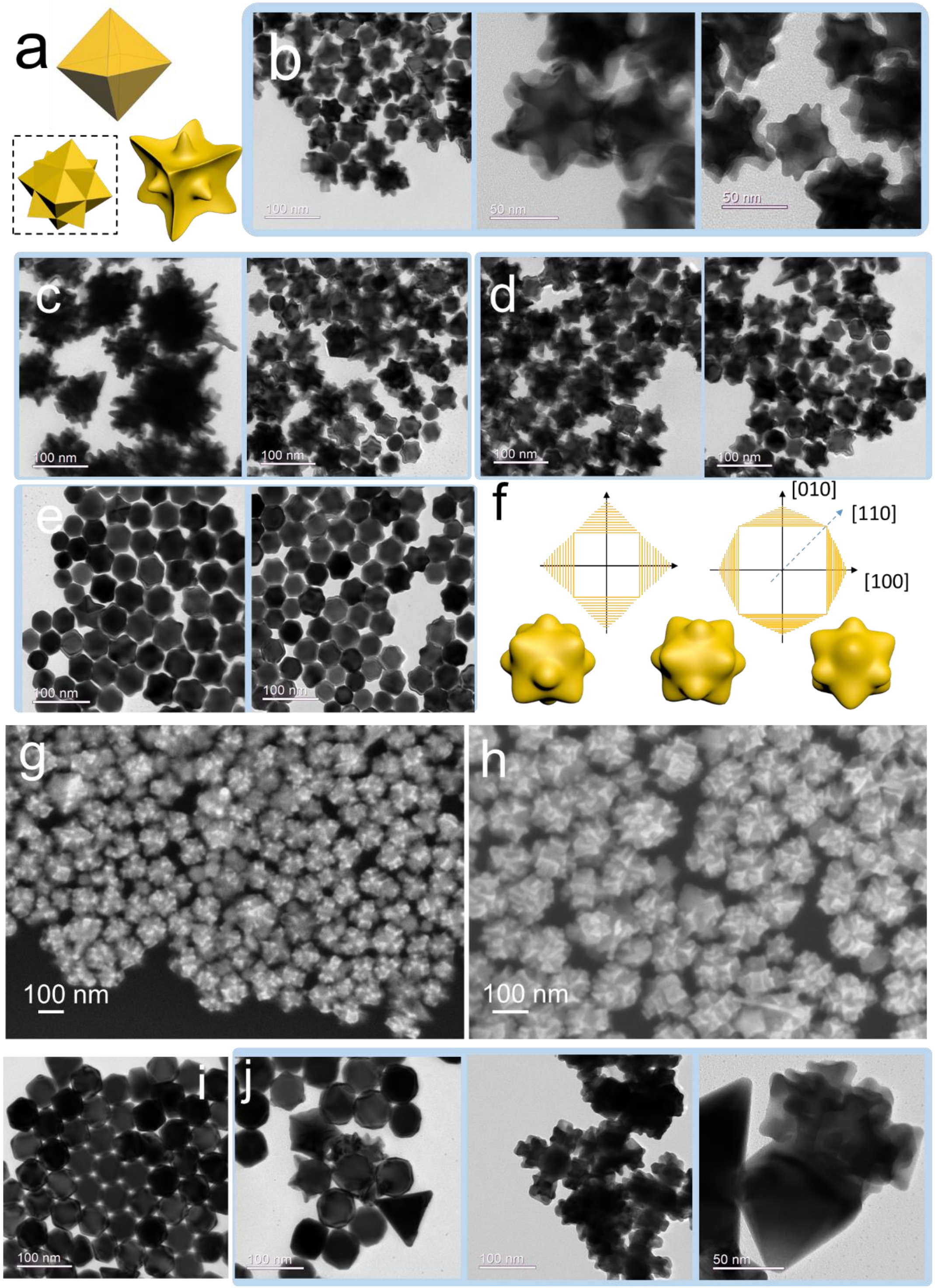

Figure S16. Different branching structures based on Au octahedra may evolve depending on the synthesis conditions.

The octahedra used here are the same as in Figure S9c1.

(a) There are no singular sites on the surface of octahedral seeds. However, during the layer advancement to obtain high-index facets (according to the growth conditions) with octahedral seeds, an intermediate cube-like structure would be formed, which could provide positions for branching. The formation of such a structure is

similar to the situation depicted in Figure S5g-i. The propagation and dissipation of kinematic waves could automatically lead to a structure conforming to the growth environment (f).

(b-h) Accordingly, some protrusions could be formed. (b, d) The amount of seeds used here is 10 µL. (c) The amount of seeds used here is 2 µL.

(e) TEM images of the intermediate cube-like structures obtained during the branching process of the octahedra. The amount of seeds used here is 20 µL. The amount of AA (0.5M) used here is 80 µL and 40 uL, respectively.

(f) 2D sketch of a cross-section of the evolution of the intermediate structure and 3D structures of the intermediate structure.

(g, h) SEM images of (d, c), respectively

(i, j) When the octahedron size was large (i), such intermediate structure was not formed, and branching at corners would occur.

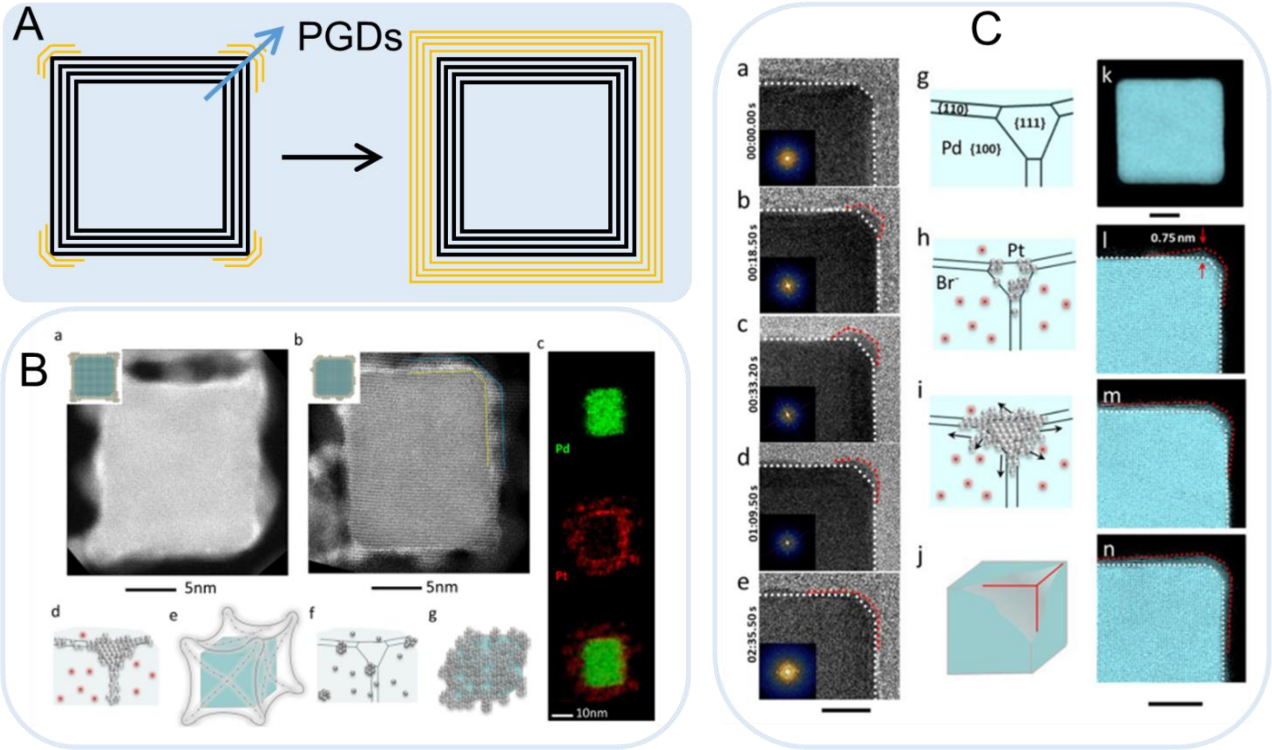

Figure S17. scheme and reported *in situ* results with PGDs of <111> directions

(A) If the PGDs are <111> directions, the protrusions would be flattened by kinematic waves to form a larger cube.
(B, C) The predicted growth was well confirmed by a reported *in situ* growth of Pt on Pd cubes by liquid cell TEM (LC-TEM). Depositing Pt on Pd cubes allows the easier visualization of growth. It has been found that the PGDs of Pt in these conditions were <111>[11]. That is to say, atoms would preferentially deposit at corners. In the low precursor concentration test (C), the nucleation process at corners was clearly shown (C b, d), and the layer advancement to enlarge the particle was also observed (C e, l, m, n). These results confirmed the predictions made at (A). In the high precursor concentration test (B), the energy barriers of surface nucleation at other sites could be overcome. Thus, nucleation events could occur there (B b). However, the overall shape would still show PGDs of <111> (B a, e). Adapted with permission from Ref. [12]. Copyright 2021, Springer Nature (Nat Commun 2021, 12, 3215)

References

[1] G. González-Rubio, V. Kumar, P. Llombart, P. Díaz-Núñez, E. Bladt, T. Altantzis, S. Bals, O. Peña-Rodríguez, E. G. Noya, L. G. MacDowell, A. Guerrero-Martínez, L. M. Liz-Marzán, *ACS Nano* **2019**, *13*, 4424-4435.

[2] W. Kaminsky, *J. Appl. Crystallogr.* **2007**, *40*, 382-385.

[3] M. J. Lighthill, G. B. Whitham, *Proceedings of the Royal Society of London. Series A. Mathematical Physical Sciences* **1955**, *229*, 317-345.

[4] W.-K. Burton, N. Cabrera, F. Frank, *Philosophical Transactions of the Royal Society of London. Series A, Mathematical Physical Sciences* **1951**, *243*, 299-358.

[5] F. Frank, *Z. Phys. Chem.* **1972**, *77*, 84-92.

[6] E. Carbó-Argibay, B. Rodríguez-González, S. Gómez-Graña, A. Guerrero-Martínez, I. Pastoriza-Santos, J. Pérez-Juste, L. M. Liz-Marzán, *Angew. Chem.* **2010**, *122*, 9587-9590.

[7] H. Katz-Boon, C. J. Rossouw, M. Weyland, A. M. Funston, P. Mulvaney, J. Etheridge, *Nano Lett.* **2011**, *11*, 273-278.

[8] T. Ming, W. Feng, Q. Tang, F. Wang, L. Sun, J. Wang, C. Yan, *J. Am. Chem. Soc.* **2009**, *131*, 16350-16351.

[9] M. J. Walsh, W. Tong, H. Katz-Boon, P. Mulvaney, J. Etheridge, A. M. Funston, *Acc. Chem. Res.* **2017**, *50*, 2925-2935.

[10] F. Kirner, P. Potapov, J. Schultz, J. Geppert, M. Müller, G. González-Rubio, S. Sturm, A. Lubk, E. Sturm, *Journal of Materials Chemistry C* **2020**, *8*, 10844-10851.

[11] S. Xie, S.-I. Choi, N. Lu, L. T. Roling, J. A. Herron, L. Zhang, J. Park, J. Wang, M. J. Kim, Z. Xie, *Nano Lett.* **2014**, *14*, 3570-3576.

[12] W. Gao, A. O. Elnabawy, Z. D. Hood, Y. Shi, X. Wang, L. T. Roling, X. Pan, M. Mavrikakis, Y. Xia, M. Chi, *Nat. Commun.* **2021**, *12*, 3215.
